# An Enhanced Virtual Force Algorithm for Diverse *k*-Coverage Deployment of 3D Underwater Wireless Sensor Networks

**DOI:** 10.3390/s19163496

**Published:** 2019-08-09

**Authors:** Wenming Wang, Haiping Huang, Fan He, Fu Xiao, Xin Jiang, Chao Sha

**Affiliations:** 1 College of Computer, Nanjing University of Posts and Telecommunications, Nanjing 210023, China; 2 University Key Laboratory of Intelligent Perception and Computing of Anhui Province, Anqing Normal University, Anqing 246011, China; 3 Jiangsu High Technology Research Key Laboratory for Wireless Sensor Networks, Nanjing 210023, China

**Keywords:** diverse *k*-coverage, sensor networks, three-dimensional coverage, underwater sensor networks, virtual force algorithm

## Abstract

The combination of Wireless Sensor Networks (WSNs) and edge computing not only enhances their capabilities, but also motivates a series of new applications. As a typical application, 3D Underwater Wireless Sensor Networks (UWSNs) have become a hot research issue. However, the coverage of underwater sensor networks problem must be solved, for it has a great significance for the network’s capacity for information acquisition and environment perception, as well as its survivability. In this paper, we firstly study the minimal number of sensor nodes needed to build a diverse *k*-coverage sensor network. We then propose a *k*-Equivalent Radius enhanced Virtual Force Algorithm (called *k*-ERVFA) to achieve an uneven regional coverage optimization for different *k*-coverage requirements. Theoretical analysis and simulation experiments are carried out to demonstrate the effectiveness of our proposed algorithm. The detailed performance comparisons show that *k*-ERVFA acquires a better coverage rate in high *k*-coverage sub-regions, thus achieving a desirable diverse *k*-coverage deployment. Finally, we perform sensitivity analysis of the simulation parameters and extend *k*-ERVFA to special cases such as sensor-sparse regions and time-variant situations.

## 1. Introduction

The research on Underwater Wireless Sensor Networks (UWSNs) has a wide application prospect in marine hydrological data collection, marine pollution detection, water quality monitoring [1,2,3], etc. The coverage optimization of UWSNs has a great significance for the network’s capacity for information acquisition and environment perception, as well as its survivability/lifetime [4]. In addition, the coverage effectiveness of UWSNs has a direct bearing on the properties of the network, such as its communication bandwidth and computation capacity, thus determining the quality of service to a certain extent [5].

Existing research on coverage optimization mainly aims at ground sensor networks and can be classified into three categories: the target coverage [6], the regional coverage [7], and the barrier coverage [8,9,10]. The target coverage requires that sensor networks can monitor target nodes. In regional coverage, any point inside the region must be covered by at least one active node. The barrier coverage studies the probability of detecting moving objects when they pass through the monitoring region. In [6,7,10,11,12], a variety of coverage control methods were proposed, among which the Virtual-Force Algorithm (VFA) by Zou et al. [7] has attracted considerate attention and is regarded as an effective method to solve the coverage problems in two-dimensional sensor networks. In practical applications, only some parts of the whole underwater area will be the Regions Of Interest (ROI). Observers usually expect the ROIs to be covered by more sensors (i.e., *k*-coverage) instead of only one sensor (i.e., one-coverage), so that more reliable and accurate sensing data of ROIs can be collected for further processing. In view of this, we define diverse *k*-coverage as the coverage optimization problem in which the multiplicity of *k*-coverage requirements in different regions is diverse. However, most of the algorithms above aim to solve the one-coverage problem rather than the *k*-coverage problem of ROIs, even if the *k*-coverage (k≥ 2) requirement is more practical in regional coverage of UWSNs.

In addition, typical coverage optimization utilizes a cloud-based centralized architecture. The data generated by underwater nodes is preprocessed and subsequently sent to cloud servers. The server can potentially receive huge data volumes from the large number of underwater nodes and generate significant load on the sensor network. This process will consume many computational resources and generate a certain time delay, which has a significant impact on coverage optimization. Edge computing [13,14,15], a new paradigm that adds additional edge computing servers to low-powered devices and networks, plays an important role in coverage optimization because of its advantages in agility, intelligence, reliability, and real-time performance [16,17,18]. Tasks of UWSNs can be fully or partly uploaded to the edge servers, and then, the edge servers return the computational results to the nodes for optimizing the coverage. For example, the anchor node can adjust the location of the node in real time according to the calculation result of edge servers. Meanwhile, few researchers have applied the virtual force algorithm to the diverse *k*-coverage problems of UWSNs. Therefore, this motivates us to design an effective diverse *k*-coverage algorithm based on VFA with a new computing architecture.

The main contributions of this paper are summarized as follows.

- We analyze and derive the minimal number of sensor nodes needed to build a specific diverse *k*-coverage UWSN.
- We design an enhanced virtual force algorithm *k*-ERVFA to solve the non-uniform *k*-coverage optimization problem of sub-regions with different interest levels in UWSNs.
- We extend our proposed algorithm to special cases such as sensor sparse regions and time-variant situations.

The rest of this paper is organized as follows: Section 2 summarizes related studies on sensor deployment methods of UWSNs and the *k*-coverage problem of WSN. In Section 3, we introduce the 3D-UWSNs model with different *k*-coverage requirements. We then derive the minimal number of nodes needed to meet the specific *k*-coverage requirement. In Section 4, we give a detailed description of the *k*-ERVFA algorithm. Simulation results are given in Section 5. In Section 6, we give discussions about simulation parameters and extend *k*-ERVFA to special cases. Section 7 concludes the whole paper.

## 2. Related Works

### 2.1. Virtual Force Algorithm in Wireless Sensor Network

The VFA has attracted considerable attention and is considered an effective method to solve the coverage problems. For example, Zou et al. [7] achieved the coverage control in two-dimensional sensor networks based on VFA, which moves the node according to the calculated position in each step. In this algorithm, a node will be subject to three resultant forces (obstacles, neighbor nodes, and targets to be covered), including gravity and repulsion. Tao et al. [19] proposed PFCEA (Potential Field-based Coverage-Enhancing Algorithm) based on the rotatable directional sensing model. Furthermore, Huang et al. [20] put forward node re-deployment algorithm PRMCA (Probability model based Rotate or Move along a fixed direction Coverage-enhancing Algorithm) based on the probability model and the virtual force, where the sensor node rotates or moves along the fixed direction in a two-dimensional plane in order to achieve the coverage effect. Heo et al. [21] suggested a coverage control algorithm IDCA (Intelligent Deployment and Clustering Algorithm), and the virtual force between nodes is represented by the result of the expected deployment density divided by the current deployment density. Ma et al. [22] proposed a coverage-control VFA-ACE (Area Coverage Enhance) algorithm based on the virtual potential field, aiming at three-dimensional directional sensor networks. Tan et al. [23] proposed a three-dimensional space deployment algorithm applied to continuous target tracking. Virtual force in this algorithm is generated by the inter-node force, the obstacle repulsive force, the monitored-path attractive force, and the tracking target together.

### 2.2. Existing Studies on k-Coverage of Wireless Sensor Networks

The *k*-coverage problem of wireless sensor networks has been extensively studied and can be divided into two categories: the two-dimensional cases and the three-dimensional cases. Huang et al. [24] studied two-dimensional *k*-coverage with geometric analysis and achieved improved coverage. Wan et al. [25] derived the upper and lower bound of the minimal density of nodes in the two-dimensional *k*-coverage problem using the methods based on the probability model. The studies mentioned above focused on the thick deployment regions, such as a square or a circular area. With finite thin strip regions, Balister et al. [26] calculated the deployment density of sensor nodes needed to maintain both network connectivity and the *k*-coverage property. Qiu et al. [27] proposed a distributed cooperation scheme based on a local *k*-order Voronoi diagram in which nodes cooperate in hole detection and recovery. Esnaashari et al. [28] proposed CLA-EDS (a Cellular Learning Automata-based Enhanced Deployment Strategy), a modification of the CLA-DS (a Cellular Learning Automata-based Deployment Strategy) [29], in which a learning algorithm is applied to meet the time-variant and diverse *k*-coverage requirements in the two-dimensional cases. Yu et al. [30] studied the *k*-coverage problem in WSNs and proposed protocols based on the coverage contribution area. The proposed protocols achieved low sensor spatial density and prolonged the network lifetime.

For three-dimensional *k*-coverage problems, Alam and Haas [31] proposed a deployment strategy based on the Voronoi diagram on the tessellation of the truncated octahedral in 3D space. However, *k*-coverage was not studied in [31], and only one-coverage was guaranteed. Huang et al. [32] developed a polynomial time algorithm to solve the α-coverage problem in three-dimensional sensor networks. Ammari and Das [33,34] designed a distributed hybrid forwarding protocol, which guaranteed *k*-coverage, and discussed the minimal deployment density of sensor nodes required in the three-dimensional *k*-coverage problem. In [35], a localized, pseudo-distributed scheme was further proposed to achieve *k*-coverage in 3D duty-cycled WSNs, but the diverse *k*-coverage requirements in different sub-regions were not mentioned.

### 2.3. The Existing Deployment Methods of UWSNs

The existing deployment methods of three-dimensional underwater sensor networks can be classified into two categories: the sea-bottom deployment methods and the sea-column deployment methods. Table 1 clearly displays the deployment classification of UWSN.

In the sea-bottom deployment, the seabed plane under the monitoring area is divided into many triangular grids, with nodes deployed in the intersections of the grids, so as to achieve full coverage of the monitoring area with a minimum number of nodes. Due to the 3D feature of the underwater environment, most applications require the network to collect subaqueous or aquatic data. Therefore, it is hard for the sea-bottom deployment methods to meet such demands.

In the sea-column deployment, nodes are deployed into 3D underwater space in order to achieve 3D coverage of the monitoring area. The existing sea-column deployment strategies fall into two categories: the uniform deployment and the non-uniform deployment. The uniform deployment requires the sensor nodes to be distributed uniformly in the monitoring area. Many uniform coverage optimization methods [36,37,38] have been proposed by continuously adjusting the diving depth of nodes and reducing sensing overlap areas of adjacent nodes. Alam et al. [31] proved the optimality of the truncated octahedron model in the problem of the three-dimensional coverage of sensor networks and provided various deployment strategies for practical applications. However, the coverage requirements of many practical applications are non-uniform. Some sub-regions of the whole monitoring area need to be covered by more than one sensor node; therefore, the uniform deployment-oriented optimization algorithms cannot achieve a satisfactory node deployment.

The non-uniform deployment strategy, on the other hand, allows sensor nodes to be deployed non-uniformly according to the distribution states of underwater targets. Many research efforts working on the non-uniform deployment strategy are based on the concept of the “target interest event”, which means the purpose of node deployment is to cover the targets and interesting events instead of the whole monitoring area [39] and to make the density distribution of sensor nodes be consistent with that of the target events. The non-uniform deployment strategy is more effective in practical applications and accords with the sparsity characteristic of sensor nodes deployed in UWSNs [6,21]. Aitsaadi et al. [40] proposed DDA (Differentiated Deployment Algorithm) for water quality monitoring in a closed lake. The algorithm took the distribution characteristics of pollutants into consideration and deployed nodes non-uniformly using mesh grid representation, thus achieving diverse coverage of the monitoring area. Golen et al. [41] divided the monitoring area into sub-regions based on environmental factors such as acoustic characteristics and then optimized node deployment using the Game Theory Field Design (GTFD) model. However, this method is difficult to implement due to its high complexity. Zakia et al. [42] proposed a heuristic deployment strategy based on sub-cube tessellation and mixed integer linear program optimization. Liu et al. [43] studied the topology control of UWSNs with diverse coverage requirements and proposed two algorithms in which the sensing radius of a sensor node is adjusted to achieve the diverse coverage requirements. Wang et al. [44] proposed a self-deployment algorithm for maintaining the maximum coverage and connectivity in underwater acoustic sensor networks based on an ant colony optimization in 2019. They carried out the greedy strategy and improved the path selection probability and pheromone update system.

However, current non-uniform deployment strategies based on event-driven cannot fully meet the demands of underwater sensor network applications. The main reasons are listed as follows:

- Most of the event-driven coverage algorithms obtained only one-coverage in the target event area, which might not meet the demands of practical applications. For instance, target localization tasks require that any point within the region must be covered by at least three sensor nodes.
- Many methods mainly considered determined target events while ignoring the uncertain events in the open underwater environment. Not much attention was paid to the difference of the levels of coverage required in different sub-regions. Some research works considered diverse *k*-coverage requirements in different sub-regions, but only in two-dimensional situations [28], which needed to be extended to three-dimensional cases.

Current research works covered some aspects of the *k*-coverage problems in three-dimensional space, but few methods have been proposed to solve the diverse *k*-coverage problem in UWSNs. Some solutions for UWSNs were only extended from the general 3D *k*-coverage cases. Therefore, we propose an enhanced virtual force algorithm *k*-ERVFA (*k*-Equivalent Radius enhanced Virtual Force Algorithm) to solve effectively the diverse *k*-coverage problem in 3D UWSNs. The algorithm is based on the concept of “*k*-virtual force”, and it studies the minimum deployment density of sensors required for 3D UWSNs’ *k*-coverage according to the practical application. The proposed algorithm can realize different *k*-coverage requirements in 3D UWSNs and can be extended to general situations, such as the cases of node sparsity and *k*-coverage time-varying requirements.

## 3. 3D UWSNs’ Network Model with the Diverse k-Coverage Requirement

### 3.1. Preliminaries and Problem Statement

In this paper, the proposed network model consists of anchors, underwater sensor nodes, and buoy nodes, which are connected to the sensor nodes via a rigid cable. Our goal is to achieve a desirable diverse *k*-coverage rate with minimal adjustment cost. We made the following assumptions:

1. The 3D underwater space *A* is a cube with side length $L_{0}$ (expressed as $A={L_{0}}^{3}$), and its bottom surface is the plane of the seafloor with the X-Y axis, while the *Z*-axis extends forward to the sea surface. The $i^{\mathrm{th}}$ sub-region with the *k*-coverage requirement is denoted by $A_{k,i}$. The volume of $A_{k,i}$ is denoted as $V_{A_{k,i}}$, and $A_{k,i}$ should be *k*-covered. Both $V_{A_{k,i}}$ and *k* are known in advance.
2. At the initial phase, a fixed number of sensor nodes and buoy nodes are sprinkled randomly by an airplane. The coordinates of sensor nodes are randomly distributed. The buoy nodes are fixed through anchors on the seafloor so that the (x,y) coordinates of the sensor nodes are also fixed. The diving depth of the sensor nodes can be adjusted by stretching the rigid cable between the sensor nodes and buoy nodes. Each underwater sensor node $S_{i}$ communicates with its buoy node $B_{i}$ via a wired cable and reports its current depth, and the perception data are collected. Each buoy is equipped with a GPS module in order to obtain its coordinate. In addition, the rigid wired cable guarantees that the buoy will not drift far.
3. The sink nodes collect the coordinates of buoy nodes and the corresponding underwater sensor nodes, then run the re-deployment algorithm, which calculates the re-deployment positions (only in the vertical direction) of sensor nodes to meet the *k*-coverage requirements of the sub-regions. When the calculation is complete, the sink nodes send a re-deployment message to the buoy nodes, and the buoy nodes adjust the length of the underwater wired cable; thus, the diving depths of sensor nodes are adjusted, and re-deployment is implemented.
4. Assume that the satisfactory *k*-coverage rate is denoted by η, whose value needs to be determined by practical applications, while a fixed value of the *k*-coverage rate is necessary for the more concise theoretical derivation and experimental simulations in this paper. In order to make the analysis more intuitive, the value of η will be set to 89% as a demonstration in this paper. Note that the input parameter η is still optional and adjustable. The proposed model and algorithm of this paper can adapt to different η values to meet various requirements.

Under the above assumptions, the proposed algorithm will calculate the number of sensors needed for a given diverse *k*-coverage problem and adjust the coordinates of sensor nodes automatically to achieve the required coverage rate. The explanations of the main notations are presented in Table 2.

### 3.2. Related Definitions of the Network Model

**Definition 1** (Sub-regions with different *k*-coverage requirements)**.** *The whole underwater monitoring area consists of q sub-regions of various levels of coverage requirements. Namely, for any sub-region $A_{k,i}$(i=1,2,…,q, $k\in Z^{+}$), any point inside $A_{k,i}$ should be at least k-covered.*

**Definition 2** (Perceptual model)**.** *The Boolean perceptual coverage model is adopted in this paper. Let r be the coverage radius of the sensor node and ($x_{i},y_{i},z_{i}$) be the coordinates of sensor node $S_{i}$. Then, the region covered by $S_{i}$ is a three-dimensional sphere with a radius of r and the center at ($x_{i},y_{i},z_{i}$). The region is called the perceptual sphere region, denoted as ε(i). Namely, it is the set of all points p that conform to Equation (1), in which $d(S_{i},p)$ denotes the Euclidean distance between $S_{i}$ and p.*

(1) $$\varepsilon \left(i\right)=\{p|d\left(S_{i},p\right)\leq r\}$$

**Definition 3** (*k*-equivalent radius)**.** *Let r be the coverage radius of the sensor node. Its k-equivalent radius is then defined as:*

(2) $$r_{k}=\frac{r}{k}\;\;k=1,2,3\ldots$$

**Definition 4** (*k*-conflict radius)**.** *In the classic virtual force algorithm, two sensor nodes with Euclidean distance less than 2r are considered as conflicting nodes. Similarly, we consider two nodes to be k-conflicting nodes when the Euclidean distance between them is less than two-times the k-equivalent radius. The k-conflict radius $r_{conf,k}$ is then defined in Equation (3):*

(3) $$r_{conf,k}=2r_{k}$$

**Definition 5** (*k*-coverage rate)**.** *With sub-region $A_{k,i}$, we define its k-coverage rate (denoted as $C_{k,i}$) as the volume of the k-covered region within $A_{k,i}$ divided by the total volume of $A_{k,i}$:*

(4) $$\begin{array}{c} C_{k,i}=\frac{V_{k}\left(p_{k,i}\right)}{V_{A_{k,i}}}=\frac{\int_{A_{k,i}}x_{k}\left(p_{k,i}\right)dp_{k,i}}{V_{A_{k,i}}}\;\;k=1,2,3\ldots \\ p_{k,i}\in A_{k,i},\Delta \left(p_{k,i}\right)\to 0\\ x_{k}\left(p_{k,i}\right)=\left\{\begin{array}{c} 1\;\;\;\mathrm{if}\;p_{k,i}\;\mathrm{is}\;\mathrm{k-covered}\\ 0\;\;\;\mathrm{otherwise} \end{array}\right. \end{array}$$

*where $\Delta (p_{k,i})$ is the volume of point $p_{k,i}$, which reflects the granularity of division.*

**Definition 6** (Sphere with the *k*-equivalent radius)**.** *The three-dimensional sphere with a radius of $r_{k}$ ($r_{k}=\frac{r}{k},k=1,2,3\ldots$) is defined as a sphere with the k-equivalent radius. Note that the actual physics coverage radius is still r.*

### 3.3. Analysis of the Deployment Density Satisfying Different k-Coverage Requirements

In this section, we consider the deployment density needed to meet the specific *k*-coverage requirement in a sub-region of UWSNs. Notice that [33,34] studied the minimal deployment density to meet the *k*-coverage requirement in 3D underwater region. In this paper, we obtain an improved minimum volume density of sensor nodes compared to that in [33,34]. The result is as follows:

**Claim** **1.** *The minimum volume density of sensor nodes needed to obtain a desirable k-coverage rate (89%) is denoted as $\rho_{kmin}$, and the value of $\rho_{kmin}$ is $3\sqrt{3}/8r^{3}$, when k= 1; $3/\pi r^{3}$, when k = 2; $9/2\pi r^{3}$, when k = 3... (for other k values, refer to Equation (13)).*

**Figure 1:**
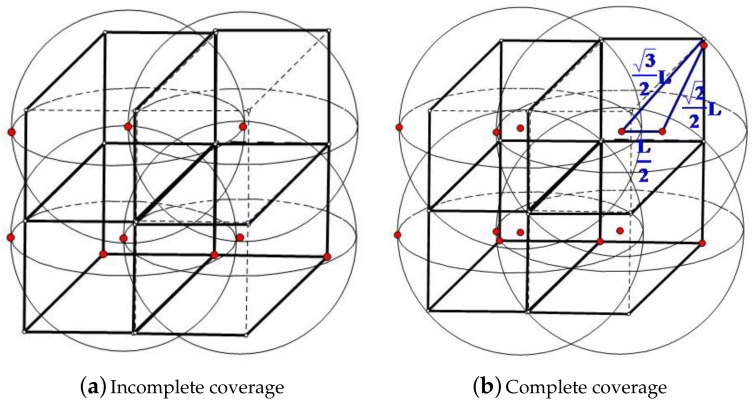
Coverage of a cubic grid. (**a**) Incomplete coverage; (**b**) Complete coverage.

To reach the above conclusion, firstly, we divide the 3D underwater space into cubic grids as shown in Figure 1a (four grids are illustrated here). Let *L* be the side length of the small cubic grid and *r* be the coverage radius of the sensor node. Each sensor node is placed in the center of the corresponding cubic. We can see from Figure 1a that if the eight vertices are not covered by the centered sensor, it will not be covered by sensors from other grids either. In this case, similar coverage breaches will occur in every cubic grid in the 3D space. Namely, when *r* is not large enough (compared to *L*), the coverage rate will not be satisfactory. However, when *r* increases to the case shown in Figure 1b, all eight vertices of the cubic grid will be covered. Thus, in order to ensure a 100% one-coverage rate, we have:

(5) $$r_{\min}=\frac{\sqrt{3}}{2}L,\;namely\;r\geq r_{\min}=\frac{\sqrt{3}}{2}L$$

For the convenience of the solving process, *L* is represented as $L=\frac{2r}{m\cdot \sqrt[{3}]{k}}$, where *k* is the coverage multiplicity required in this given sub-region and *m* is an unknown parameter. According to the discussion above, we have:

(6) $$r\geq r_{\min}=\frac{\sqrt{3}}{2}L=\frac{\sqrt{3}r}{m\cdot \sqrt[{3}]{k}},\;m\geq \frac{\sqrt{3}}{\sqrt[{3}]{k}}$$

Sensor nodes are deployed uniformly in the whole 3D underwater space, so the density of sensor nodes is:

(7) $$\rho =\frac{1}{L^{3}}=\frac{1}{{\left(\frac{2r}{m\cdot \sqrt[{3}]{k}}\right)}^{3}}=\frac{m^{3}k}{8r^{3}}$$

Consider some point *p* inside the given sub-region and a sphere centered at *p* with a radius of *r*; the number of sensor nodes inside the sphere is:

(8) $$n=\frac{4}{3}\pi r^{3}\cdot \rho =\frac{4}{3}\pi r^{3}\cdot \frac{m^{3}k}{8r^{3}}=\frac{m^{3}\pi k}{6}$$

Any sensor *S* inside the sphere covers point *p*, while for any sensor $S^{\prime }$ outside this sphere, the Euclidean distance between $S^{\prime }$ and *p* is greater than *r*, which means that *p* will not be covered by $S^{\prime }$. In order to meet the *k*-coverage requirement, there shall be at least *k* sensors inside this sphere; thus, we have:

(9) $$n\geq k,\;namely\;\frac{m^{3}\pi k}{6}\geq k,\;m\geq \sqrt[{3}]{\frac{6}{\pi }}=1.2407$$

Theoretically, *k*-coverage requirement will be met when both Equations (6) and (9) are satisfied simultaneously. However, the analysis above mainly considers the average number of sensors. In practice, the 100% *k*-coverage rate requirement cannot always be met due to randomness in sensor deployment.

**Figure 2:**
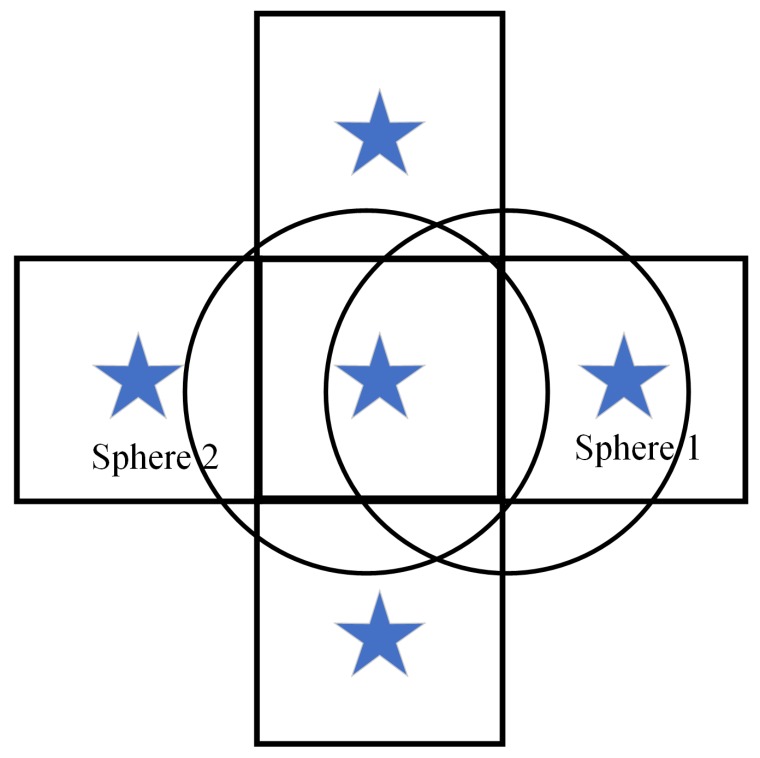
A counter-example to average volume.

For example, as shown in Figure 2, which is the 2D projection of the 3D case mentioned above, blue pentagrams stand for sensor nodes. Sphere 1 and Sphere 2 have the same volume, but Sphere 1 contains two sensor nodes, while Sphere 2 contains only one sensor node. Namely, all particles inside Sphere 1 are two-coverage, while the particles inside Sphere 2 and outside Sphere 1 are one-coverage.

Therefore, an adjustable parameter θ is introduced to ensure higher *k*-coverage in the actual deployment, which makes our solution more suitable for random deployment of initialization.

Therefore, the above condition $\frac{m^{3}\pi k}{6}\geq k$ should to be adjusted to:

(10) $$\frac{m^{3}\pi k}{6}\geq \theta k\Rightarrow m\geq \sqrt[{3}]{\frac{6\theta }{\pi }},\;\theta >1$$

where θ is introduced to provide some redundancy of nodes so that a high *k*-coverage rate can be guaranteed. θ is related to both *k* and η, expressed as θ=θ(k,η). The value of θ should be carefully chosen, for a higher θ value will lead to a higher sensor deployment cost, while the satisfactory coverage rate will not be obtained if θ is too small. We propose the Optimal θ Searching Algorithm (OθSA) to help decide the appropriate value of θ.

In this paper, the coverage radius of sensor node *r* = 10 m, and the side length of the 3D underwater region $L_{0}$ = 100 m. Sensor nodes were deployed as shown in Figure 1, with a deployment interval of $L=2r/(m\cdot \sqrt[{3}]{k})$. In order to make the analysis more intuitive, the value of η will be set to 89% as a demonstration in this paper.

**Figure 3:**
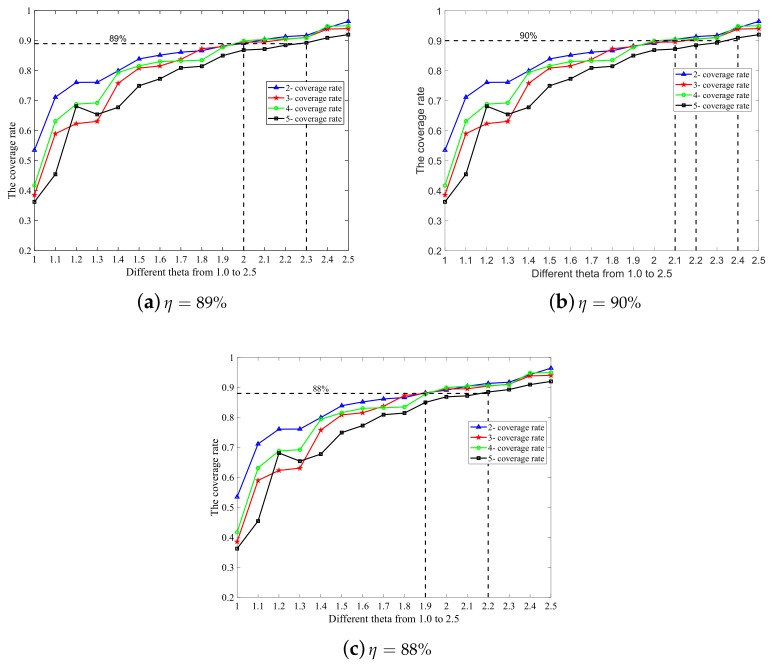
The simulation results of the coverage rate with different values of *k* and *θ*(*k, η*). (**a**) *η* = 89%; (**b**) *η* = 90%; (**c**) *η* = 88%.

In order to meet different requirements of the *k*-coverage rate in practical applications, we can obtain the optimal θ value through the optimal θ searching algorithm (Algorithm 1). When η is varied, the new θ value can be calculated by this algorithm to match it. In addition, we have displayed different values regarding θ when η = 88% and 90%, respectively, as shown in Figure 3 and Table 3 below.

**Algorithm 1:** Optimal θ searching algorithm.

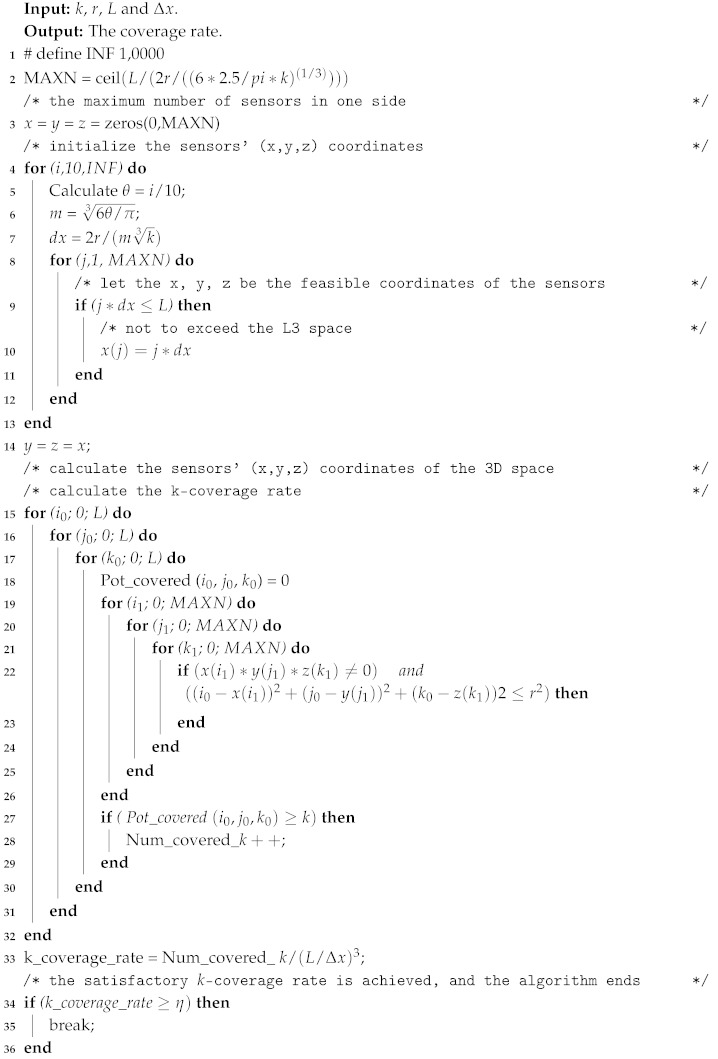



In the simulation experiment, we set the step size of spatial variation Δx to be one meter. We performed the search with different *k* values and varied θ(k,η) from 1.0–2.5 with an increment of 0.1. Simulation experiments were carried out several times, and the average coverage rate was calculated to decide the appropriate θ(k,η). The simulation results are shown in Figure 3.

As we can see from Figure 3b, when θ(k,η) = 1, which is the aforementioned case without any redundancy of nodes, the two-coverage rate is only 53.47%, and 38.52% for three-coverage, 41.68% for four-coverage, and 36.23% for five-coverage. The coverage rate increases significantly when θ grows. The optimal θ to achieve the 89% *k*-coverage rate is as follows: θ(2, 89%) = θ(3, 89%) = θ(4, 89%) = 2, and θ(5, 89%) = 2.3.

From Figure 3, we find that a higher value of θ (greater than 1.8) makes little contributions to the improvement of the coverage rate, while causing an unnecessary deployment cost. Thus, it is not recommended to adopt a higher value of θ. In addition, for higher values of *k*(k≥5), the optimal value of θ also should be determined by OθSA.

It can be seen in Figure 3 and Table 3 that the simulation results are consistent with the theoretical derivation. The basic idea of the algorithm can be described as follows: for a given *k*, through traversing the value of θ(k,η) from 1.0 with an increment of 0.1, the value of *m* can be calculated according to Equation (10); thus, the quantitative relation between *r* and *L* is known, and the *k*-coverage rate in the sub-region can be obtained. Whenever the calculated coverage rate reaches η, the algorithm will be terminated at this moment, and the value of θ(k,η) is optimal. This algorithm belongs to the Monte Carlo method (statistical simulation algorithm). The shorter the step size of space particle coordinates, the higher the precision and the higher the algorithm complexity. The time complexity of OθSA is $O(L_{o}^{3}MAXN^{3}/{\left(\Delta x\right)}^{3})$ where Δx is the step size of spatial variation and MAXN is ceil ($L_{0}/L$).

In summary, in order to achieve a satisfactory *k*-coverage rate of one given sub-region, both Equations (6) and (10) should be satisfied ($m\geq \frac{\sqrt{3}}{\sqrt[{3}]{k}}$ and $m\geq \sqrt[{3}]{\frac{6\theta }{\pi }}$). Besides, larger *m* leads to smaller $L=\frac{2r}{m\sqrt[{3}]{k}}$ and a greater volume density of sensor nodes, which means the deployment cost is more expensive. We should adopt the minimum *m* that satisfies both Equations (6) and (10). That is:

$$\begin{array}{c} \left\{\begin{array}{c} m\geq \frac{\sqrt{3}}{\sqrt[{3}]{k}}\\ m\geq \sqrt[{3}]{\frac{6\theta }{\pi }}\\ k\geq 1\;\&\;k\in Z^{+} \end{array}\right. \end{array}$$

The minimum *m* and corresponding volume density of nodes to ensure an 89% *k*-coverage rate can then be calculated and is shown in Table 4.

Notice that $\rho_{min}\left(r,\;5,\;89\%\right)>\rho_{min}\left(r,\;4,\;89\%\right)>\rho_{min}\left(r,\;3,\;89\%\right)>\rho_{min}\left(r,\;2,\;89\%\right)>\rho_{min}\left(r,\;1,\;89\%\right)$, which coincides with the actual situation.

In [33], the minimal volume density of sensor nodes needed to ensure *k*-coverage in 3D space is:

(11) $$\lambda \left(r,k\right)=\frac{9k}{\pi r^{3}},\;k>1$$

In [34], the volume density is:

(12) $$\beta \left(r,k\right)=\frac{k}{0.422{r_{0}}^{3}},\;k\geq 4,\;\mathrm{where}\;r_{0}=\frac{r}{1.066}$$

We will see in Section 6.2 that 1<θ(k,η)≤2.3 when η = 89% and k≥2. Therefore, we have:

(13) $$\begin{array}{cc} \rho_{\min}\left(r,k,\eta =89\%\right) & =\frac{m_{\min}^{3}k}{8r^{3}}\leq \frac{\frac{13.8}{\pi }k}{8r^{3}}\\ & =\frac{1.725k}{\pi r^{3}},\;\mathrm{when}\;k\geq 2 \end{array}$$

In comparison, λ(r,k) is several times $\rho_{min}\left(r,k,89\%\right)$, and β(r,k) is greater than $\rho_{min}\left(r,k,89\%\right)$ when k≥4, while $\rho_{min}\left(r,k,89\%\right)$ is sufficient to ensure a coverage rate of 89%. Considering the deployment cost and the achieved coverage rate, $\rho_{min}\left(r,k,89\%\right)$ has better performance than λ(r,k) and β(r,k). In practice, a one-coverage rate reaches 100% with $\rho_{min}\left(r,1,89\%\right)$ when *k* = 1, as shown in Figure 1b.

The minimal number of nodes in any given *k*-coverage sub-region can then be calculated as:

(14) $$n_{\min}\left(k,\eta \right)=V_{k}\cdot \rho_{min}\left(r,k,\eta \right)=V_{k}\cdot \frac{{m^{3}}_{\min}\left(r,k,\eta \right)\cdot k}{8r^{3}}$$

where $V_{k}$ is the volume of the *k*-coverage sub-region.

## 4. The Enhanced Virtual-Force Algorithm k-ERVFA

In this section, we propose the enhanced virtual force algorithm, i.e., *k*-ERVFA to solve the diverse *k*-coverage problem of 3D UWSNs. The model of the sensor sphere with *k*-equivalent radius (seen in Section 3.2) is used to cover the entire 3D underwater area.

### 4.1. Repulsion between k-Conflicting Nodes Based on Coulomb’s Law

According to Coulomb’s Law, we define the *k*-repulsion between two sensor nodes *i* and *j* as Equation (15):

(15) $$\vec{F_{ji}}=\left\{\begin{array}{c} \frac{K_{conf}}{d_{ij}^{2}}\cdot \vec{e_{ji}},0<d_{ij}\leq r_{conf,k}\\ 0,d_{ij}>r_{conf,k} \end{array}\right.\;i,j\in \left\{1,2,\ldots ,n\right\}$$

where *n* is the total number of sensor nodes in the sensor network, $d_{ij}$ is the Euclidean distance between sensor node *i* and sensor node *j*, $r_{conf,k}$ is the *k*-conflict radius (seen Definition 4 in Section 3.2), $\vec{e_{ji}}$ is the unit vector in the direction from $S_{j}$ to $S_{i}$, and $K_{conf}$ is the coefficient of the Coulomb repulsion between nodes, which is a constant independent of the value of *k*.

Note that for sub-regions with different *k*-values, the only difference in Equation (15) is $r_{conf,k}$, which is 2·r/k (seen in Definitions 3 and 4 in Section 3.2). For greater *k* values, both $r_{k}$ and $r_{conf,k}$ are smaller, i.e., the repulsion exists only when the Euclidean distance between neighboring nodes is rather small (compared to cases with small *k* values). A smaller distance between nodes means a higher volume density, which leads to a higher *k*-coverage rate. This explains why the *k*-equivalent radius was introduced and how it facilitates obtaining diverse *k*-coverage.

In this algorithm, the *k*-repulsion between nodes was adopted to minimize the overlapping coverage area of neighboring nodes.

### 4.2. Attraction from the k-Coverage Requirement Sub-Regions

If there were only repulsion between nodes, sensor nodes would just disperse as far as possible so there will be coverage breaches between sensors; thus, *k*-coverage will not be achieved. In an ideal deployment scheme, more sensor nodes should move towards sub-regions with *k*-coverage (k≥2) requirements to guarantee better *k*-coverage. Therefore, we introduce the attraction from the *k*-coverage requirement sub-regions on nodes to “pull in” more sensor nodes. In order to achieve diverse *k*-coverage, the attraction coefficients of sub-regions with different *k*-coverage requirements should differ. The attraction from a sub-region on nodes is considered as the attraction from the region’s centroid. For a given *k* (k≥2), the *k*-attraction from the $j^{\mathrm{th}}$*k*-coverage requirement sub-region on sensor $S_{i}$ is:

(16) $${\vec{F}}_{attr,ikj}=\left\{\begin{array}{c} \frac{K_{attr,k}}{d_{ikj}^{2}}\cdot \vec{e_{ikj}},\;\mathrm{if}\;S_{i}\;\mathrm{is}\;\mathrm{outside}\;\mathrm{of}\;A_{k,j}\\ 0,\;\;\;\;\;\mathrm{if}\;S_{i}\;\mathrm{is}\;\mathrm{inside}\;\mathrm{of}\;A_{k,j} \end{array}\right.$$

where $K_{attr,k}$ is the attraction coefficient of the *k*-coverage requirement sub-regions on sensor nodes and varies with different *k* values and $d_{ikj}$ is the equivalent distance between sensor $S_{i}$ and the *k*-coverage requirement region $A_{k,j}$ (the *j*th *k*-coverage requirement sub-region for a given *k*), which is considered as the Euclidean distance between $S_{i}$ and the centroid of $A_{k,j}$:

(17) $$d_{ikj}=\sqrt{{\left(x_{i}-x_{k,j}\right)}^{2}+{\left(y_{i}-y_{k,j}\right)}^{2}+{\left(z_{i}-z_{k,j}\right)}^{2}}$$

The direction of ${\vec{F}}_{attr,ikj}$ is determined by the unit vector $\vec{e_{ikj}}$, which starts from $S_{i}$ and ends at the centroid of $A_{k,j}$. Now, considering the value of $K_{attr,k}$, naturally, for sub-regions with higher *k*-coverage requirements, the attraction should be greater so that more sensors can be pulled into these sub-regions; thus, we will set greater $K_{attr,k}$ for higher *k* values. The numerical relation of $K_{attr,k}$ for different *k* values is determined by $\frac{K_{attr,k_{1}}}{K_{attr,k_{2}}}=\frac{k_{1}}{k_{2}}$ (the ratio of the coverage requirement multiplicity in different sub-regions), where $k_{1}$ and $k_{2}$ represent the coverage requirement multiplicity in different sub-regions, respectively. The numerical relation between $K_{attr,k}$ and $K_{conf}$ will be stated later in the simulation section.

### 4.3. Obstacle Repulsion from the “Fixed” k-Coverage Requirement Sub-Regions

The *k*-ERVFA algorithm sorts all sub-regions in a descending order of *k* values, then considers the sensor deployment in each sub-region successively. Namely, the sub-regions with high *k* values are considered as “fixed” after their deployment rounds are over. The sensor nodes inside of the “fixed” sub-regions will not participate in the future deployment process (i.e., the deployment process in sub-regions with smaller *k* values). Besides, in the following deployment process, the aforementioned “fixed” sub-regions should be considered as obstacles to prevent the nodes outside the “fixed” sub-regions from re-entering. This kind of re-entering will result in extra deployment cost and can be avoided when the obstacle *k*-repulsion is adopted.

**Figure 4:**
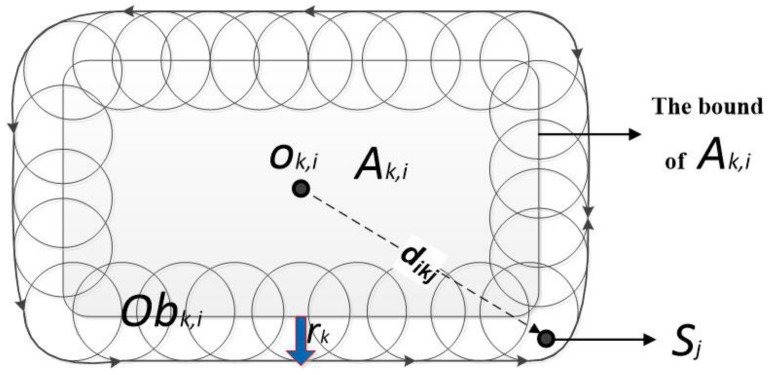
Obstacle area $Ob_{k,i}$ with the 2D form.

We set the obstacle region $Ob_{k,i}$ of “fixed” sub-region $A_{k,i}$ to be the combination of $A_{k,i}$ and a ring region formed by the spheres with a radius of $r_{k}$ and the centroid at the boundary of $A_{k,i}$, as shown in Figure 4.

$Ob_{k,i}$ can be defined as:

(18) $$Ob_{k,i}=\left\{p|d_{ob}\left(p,o_{k,i}\right)\leq d\left(p^{\prime },o_{k,i}\right)+r_{k},\;\forall p\in bond_{k,i}\right\}$$

where *p* represents a point in 3D space, $d_{ob}\left(p,o_{k,i}\right)$ is the distance from *p* to $o_{k,i}$, which is the centroid of $A_{k,i}$, $bond_{k,i}$ is the boundary surface of region $A_{k,i}$, and $p^{\prime }$ is the projection of point *p* on $bond_{k,i}$.

In the current deployment round, if sensor node $S_{j}$ is outside the fixed region $A_{k,i}$ and inside the obstacle region $Ob_{k,i}$, it will receive repulsion from $Ob_{k,i}$:

(19) $${\vec{F}}_{ob,ikj}=\left\{\begin{array}{c} \frac{K_{ob,k}}{d_{ikj}^{2}}\cdot \vec{e_{ikj}}\;\;\mathrm{if}\;S_{j}\;\mathrm{is}\;\mathrm{inside}\;\mathrm{of}\;Ob_{k,i}\;\&\&\;\;S_{j}\;\notin \;A_{k,i}\\ 0\;\;\;\;\;\;\;\mathrm{if}\;S_{j}\;\mathrm{is}\;\mathrm{outside}\;\mathrm{of}\;Ob_{k,i} \end{array}\right.$$

where ${\vec{F}}_{ob,ikj}$ represents the repulsion of obstacle region $Ob_{k,i}$ on sensor $S_{j}$ with the direction from the centroid of region $A_{k,i}$ to $S_{j}$, $d_{ikj}$ is the distance between $Ob_{k,i}$ and $S_{j}$, and $K_{ob,k}$ is the repulsion coefficient of $Ob_{k,i}$.

### 4.4. k-Resultant Force of the Sensor Node

Based on the virtual force model and the discussions above, the *k*-resultant force on node $S_{i}$ is the vector sum of the aforementioned *k*-repulsion and *k*-attraction, namely:

(20) $$\vec{F_{i}}=\sum_{j}\vec{F_{ji}}+\sum_{k,j}{\vec{F}}_{attr,ikj}+\sum_{k,j}{\vec{F}}_{ob,ikj}$$

In a single deployment round, the sink node receives the coordinate of $S_{i}$($x_{i},y_{i},z_{i}$) from the buoy, calculates the *k*-resultant force that acts on $S_{i}$, and then decides the moving direction and distance of $S_{i}$ in this round. Since the underwater sensor nodes can only move vertically, we need to obtain the component force along the *Z*-axis by projecting $\vec{F_{i}}$ onto the *Z*-axis:

(21) $$\vec{F_{iz}}=\vec{F_{i}}\cdot \frac{z_{i}}{\sqrt{x_{i}^{2}+y_{i}^{2}+z_{i}^{2}}}$$

Let $\vec{F_{i}}$ be expressed as $\vec{F_{i}}=\left(F_{ix},F_{iy},F_{iz}\right)$, then $\vec{F_{iz}}=F_{iz}\cdot \vec{e_{z}}$, where $\vec{e_{z}}$ is the unit vector in the positive direction of the *Z*-axis. The moving distance and direction of $S_{i}$ are then decided by the magnitude ($|F_{iz}|$) and direction of $\vec{F_{iz}}$. Let the maximum moving distance (along the *Z*-axis) of all the sensor nodes in a single round be $\Delta L_{max}$ (the suitable value of $\Delta L_{max}$ is discussed in Section 6). After $\vec{F_{iz}}$ of each $S_{i}$ is calculated in each round, the maximum of $|F_{iz}|$ can be obtained and be denoted by $max\{|F_{iz}|\}\;\left(i=1,2\ldots ,n\right)$. The moving distance of $S_{i}$ in this round is then:

(22) $$\Delta z_{i}=\frac{F_{iz}}{\max\{|F_{iz}|\}}\cdot \Delta L_{\max}$$

The normalized calculation in Equation (22) represents the correlation between the resultant force that acts on the sensor node and the sensor’s moving distance in each round, namely greater force corresponds to longer moving distance under the premise that the maximal moving distance in a single round is $\Delta L_{max}$.

### 4.5. Motion Pattern of Boundary Nodes Based on the Ideal Elastic Collision

Any given closed region has its boundary; here, the word “region” means either one specific *k*-coverage requirement sub-region $A_{k_{i}^{\prime },j}$ or the entire underwater monitoring area *A*. For one sub-region $A_{k_{i}^{\prime },j}$, when the “fixed and even” algorithm (seen in Section 4.6 and Section 6.1) is performed, we need to make sure that all sensor nodes inside $A_{k_{i}^{\prime },j}$ always stay in $A_{k_{i}^{\prime },j}$ (that is why these nodes are considered to be “fixed”). For the entire monitoring area *A*, naturally, any sensor node should not move outside of the boundary of *A*. To prevent nodes from escaping a closed region, we propose the motion pattern of boundary nodes based on ideal elastic collision.

In this paper, sensor nodes can only move along the *Z*-axis, so we only consider the z-coordinate. If the calculated redeployment position $z_{i}+\Delta z_{i}$ is outside the boundary, then the actual redeployment position should be modified as follows according to ideal elastic collision:

When $z_{i}=z_{i}+\Delta z_{i}>\bar{A}$:

(23) $${z_{i}}^{\prime }=\bar{A}-\left(z_{i}+\Delta z_{i}-\bar{A}\right)=2\bar{A}-\left(z_{i}+\Delta z_{i}\right)$$

When $z_{i}=z_{i}+\Delta z_{i}<\underline{A}$:

(24) $${z_{i}}^{\prime }=\underline{A}+\left(\underline{A}-\left(z_{i}+\Delta z_{i}\right)\right)=2\underline{A}-\left(z_{i}+\Delta z_{i}\right)$$

where $\bar{A}$ is the z-coordinate of the upper horizontal boundary of the 3D region *A*, $\underline{A}$ is that of the lower horizontal boundary, and ${z_{i}}^{\prime }$ is the modified redeployment position of node $S_{i}$ after collision.

### 4.6. The Fix and Even Redeployment Algorithm

In this Section, we only describe the procedure of the fix and even algorithm; a further discussion and explanation can be seen in Section 6.1.

1. The input parameter of this algorithm is the coverage multiplicity ${k_{i}}^{\prime }$. Set the state of any sensor $S_{x}$ inside $A_{{k_{i}}^{\prime },j}$ to be “fixed”. Namely, for any sensor inside $A_{{k_{i}}^{\prime },j}$, its range of motion should be within $A_{{k_{i}}^{\prime },j}$.
2. Calculate the *k*-resultant force on $S_{x}$ and its moving vector $\Delta z_{x}$, based on the discussions in Section 4.1, Section 4.2, Section 4.3 and Section 4.4. If the calculated redeployment position $z_{x}+\Delta z_{x}$ is outside $A_{{k_{i}}^{\prime },j}$, modify the actual redeployment position according to Equation (23) or (24).

Repeat Step 2 until the iteration number reaches $N_{max}$ or the *k*-coverage rate of $A_{{k_{i}}^{\prime },j}$ is met, that is $C_{{k_{i}}^{\prime },j}\geq \eta$, where η is the satisfactory *k*-coverage rate for practical application. The fix and even algorithm is described in Algorithm 2. 

**Algorithm 2:** The “fix and even” algorithm.

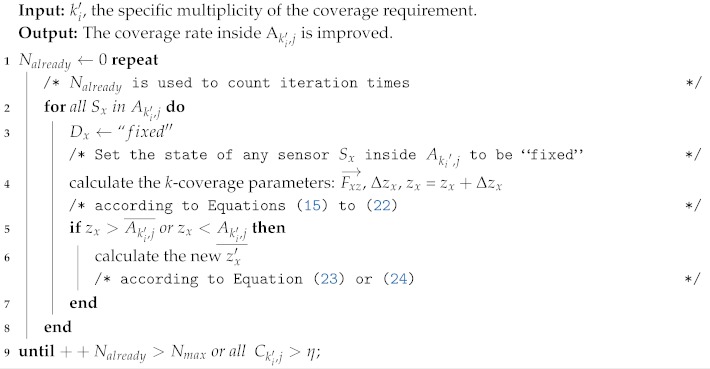



### 4.7. Steps of the k-ERVFA Algorithm

The procedure of *k*-ERVFA algorithm (described in Algorithm 3) is as follows:

1. Sort {$k_{1},k_{2}\ldots ,k_{m}$} ($k_{i}$ is the coverage multiplicity in different *k*-coverage requirement sub-regions) in descending order, and get {${k_{1}}^{\prime },{k_{2}}^{\prime }\ldots ,{k_{m}}^{\prime }$}, where ${k_{1}}^{\prime }>{k_{2}}^{\prime }>\ldots >{k_{m}}^{\prime }$. For each ${k_{i}}^{\prime }$, calculate the corresponding *k*-equivalent radius $r_{{k_{i}}^{\prime }}=r/{k_{i}}^{\prime }$.
2. Check the current iteration number $N_{already}$; if $N_{already}>N_{max}$, break out the loop in Step 2; otherwise, substitute the *k*-equivalent radius calculated in Step 1 into Equations (15)–(22). The sink nodes calculate the *k*-resultant force and the moving vector for all sensor nodes, except for those whose states are set as fixed in Step 3. The redeployment positions of sensors in this iteration round are then determined (for boundary nodes, the ideal elastic collision model will be adopted if necessary). Now, calculate the *k*-coverage rate $C_{{k_{i}}^{\prime },j}$ of $A_{{k_{i}}^{\prime },j}$, (*j* = 1,2, …, $m_{{k_{i}}^{\prime }}$, where $m_{{k_{i}}^{\prime }}$ is the total number of sub-regions that need to be ${k_{i}}^{\prime }$-covered). Break out of Step 2 if $N_{already}>N_{max}$ or the *k*-coverage rate for all $A_{{k_{i}}^{\prime },j}$ is met. Otherwise, repeat Step 2, and increase $N_{already}$ by one.
3. For all sub-regions that need to be ${k_{i}}^{\prime }$-covered, the redeployment process is partially done. Now, either the *k*-coverage rate for all $A_{{k_{i}}^{\prime },j}$ has been met or $N_{already}$ has reached $N_{max}$. Then, call the fix and even algorithm for all $A_{{k_{i}}^{\prime },j}$. By doing this, all sensors within $A_{{k_{i}}^{\prime },j}$ are set to be fixed and will no longer participate in the following redeployment rounds (smaller ${k_{i}}^{\prime }$). Besides, the sensors within $A_{{k_{i}}^{\prime },j}$ are homogenized, and a better coverage rate is achieved.
4. Increase *i* by one. If i≤m, go back to Step 2. Otherwise, the redeployment algorithm finalizes, and sink nodes send the final positions of sensors to the corresponding buoy nodes; the sensors then individually move to the calculated final positions, and the redeployment process is done.

**Algorithm 3:** The *k*-ERVF Algorithm (*k*-ERVFA).

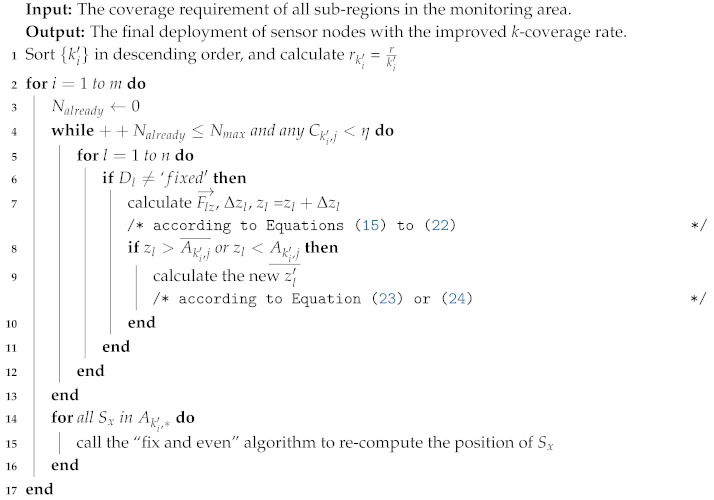



## 5. Simulation Results and Performance Analysis

We performed simulations of the *k*-ERVFA algorithm using MATLAB R2015a. The entire three-dimensional underwater monitoring area was set to be a cube with side length of 100 m. The diverse *k*-coverage requirement was set as follows:

- a cubic sub-region that is centered on (25 m, 25 m, 25 m) needs to be three-covered, and the size of the three-coverage requirement sub-region is 30 m * 30 m * 30 m;
- a cubic sub-region that is centered on (70 m, 70 m, 70 m) needs to be two-covered, and the size of the two-coverage requirement sub-region is 40 m * 40 m * 40 m;
- the rest of the monitoring area needs to be one-covered.

Sensor nodes were randomly distributed initially. We set the numerical relation of $K_{conf}:K_{attr,2}:K_{attr,3}:K_{ob,2}:K_{ob,3}$ to be 1:2:3:2:3. With a given maximum iteration number, this proportion helps to achieve a satisfactory *k*-coverage rate according to the simulation results. The detailed simulation parameters are shown in Table 5.

Here, *r* is set to be 10 m, and according to Equation (14), the minimal number of sensor nodes needed to meet the diverse *k*-coverage requirements (89% was considered as the satisfactory coverage rate) in different sub-regions is: 591 for the one-coverage sub-region; 62 for the two-coverage sub-region; 39 for the three-coverage sub-region; and 692 in total.

However, in the simulation experiments, we deployed no more than 650 sensor nodes into the monitoring area for the reasons below. Firstly, according to Equation (10) and Table 4, the calculated number of nodes will achieve a 100% one-coverage rate in the one-coverage requirement sub-regions, which is unnecessary. Secondly, θ was introduced, which leads to redundancy in node deployment. In practice, *k*-ERVFA achieved a satisfactory *k*-coverage rate with approximately 650 nodes, as shown in the simulation results.

In the simulation experiments, the number of nodes varied from 400 to 650 with an increment of 50 each time. We compared *k*-ERVFA with the following sensor deployment algorithms: RD (initial Random Deployment algorithm), VFA (classic Virtual Force Algorithm) [7], CLA-EDS (a Cellular Learning Automata-based Enhanced Deployment Strategy) [28], IDCA (Intelligent Deployment and Clustering Algorithm) [21]. Note that the IDCA algorithm does not take the *k*-coverage requirement into consideration, and it should be modified to be compared with other algorithms. We modified IDCA by replacing $d_{avg}$ (the expected distance between nodes, which was an input parameter of IDCA) with $d_{avg}/k$ (similar to the *k*-equivalent radius in *k*-ERVFA); thus, the modified IDCA can deal with diverse *k*-coverage problems. In addition, CLA-EDS and IDCA only study two-dimensional sensor networks, so we extended them to the 3D case while constraining sensor nodes to move along the *Z*-axis only. We compared five algorithms under the same conditions.

**Figure 5:**
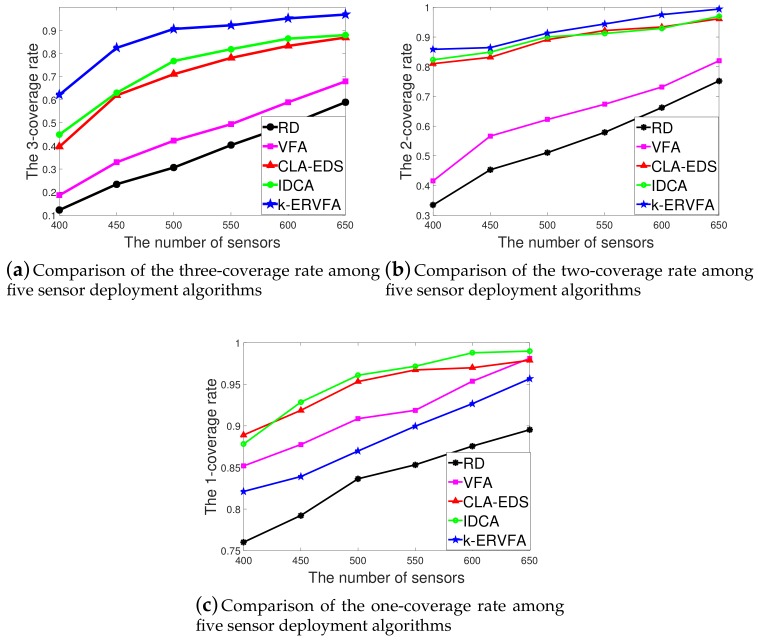
Comparison of the coverage rate among five sensor deployment algorithms. (**a**) Comparison of the three-coverage rate among five sensor deployment algorithms; (**b**) Comparison of the two-coverage rate among five sensor deployment algorithms; (**c**) Comparison of the one-coverage rate among five sensor deployment algorithms.

Figure 5a–c show the *k*-coverage rate (*k* = 3, 2, 1) achieved by different algorithms with the number of sensor nodes varying from 400 to 650.

As shown in Figure 5a, with 450 sensor nodes, for the three-coverage requirement sub-region $A_{3,1}$, the three-coverage rate was 23.43% by RD, 32.92% by VFA, 61.89% by CLA-EDS, 62.97% by IDCA, and 82.45% by *k*-ERVFA, which was the highest among the five algorithms. Similarly, in Figure 5b, with 450 sensor nodes, for the two-coverage requirement region $A_{2,1}$, the two-coverage rate was 45.32% by RD, 56.65% by VFA, 83.17% by CLA-EDS, 84.92% by IDCA, and 86.44% by *k*-ERVFA, which was also the highest. Note that although 450 was far less than 692 calculated by Equation (14), to achieve over an 89% *k*-coverage rate, the two-coverage and three-coverage rate of *k*-ERVFA reached 82% and 86%, respectively. It is obvious that *k*-ERVFA improved the *k*-coverage rate significantly.

Similar results were obtained when *n* varied from 500 to 650. The two-coverage and three-coverage rates of *k*-ERVFA were higher compared to other algorithms. It can be found that there was a significant advantage in the three-coverage rate, while the advantage was rather slight for the two-coverage rate.

Figure 5c demonstrates the one-coverage rate achieved by the five algorithms. IDCA had the highest one-coverage rate, followed by the CLA-EDS algorithm, then the classic VFA and *k*-ERVFA algorithms. Note that the *Y*-axis started at 0.75, and the difference of the coverage rate between *k*-ERVFA and IDCA was from 3.33% to 9.11% as *n* varied from 400 to 650, which shows that *k*-ERVFA increased the two- and three-coverage rate significantly with a slight sacrifice in the one-coverage rate. In addition, the one-coverage rate of *k*-ERVFA reached 91.87% when n=450, which was 12.64% higher than RD, showing that its one-coverage performance was acceptable.

The main reason was that the *k*-ERVFA algorithm sorted all diverse *k*-coverage requirement sub-regions in terms of *k* values and considered redeployment in high *k*-coverage sub-regions preferentially. Namely, the algorithm deployed nodes into high *k*-coverage requirement sub-regions first, followed by the redeployment in low *k*-coverage sub-regions. In particular, for some point in a sub-region that needs to be *k*-covered, if it can only be (k−1) covered because of the lack of one sensor node, then the other existing (k−1) sensor nodes are wasted for this point. For higher *k* values, this kind of squander of sensors was severe and should be eliminated with first priority, which explains why *k*-ERVFA set priorities according to the *k* values.

In this respect, *k*-ERVFA was superior to CLA-EDS, which did not take priority coverage for the higher *k*-coverage requirement into consideration (according to [28], the CLA-EDS algorithm showed an obvious decrease in the high *k*-coverage rate). IDCA introduced the *k*-expected distance $d_{avg}/k$, which was similar to the *k*-equivalent radius in *k*-ERVFA, and both algorithms were based on VFA, while the *k*-ERVFA algorithm achieved a better two- and three-coverage rate by considering the *k*-resultant force comprehensively and applying the fix and even algorithm to redeploy nodes within sub-regions. Compared to the classic VFA algorithm in which nodes are deployed uniformly, *k*-ERVFA pulled more nodes into the two- and three-coverage requirement sub-regions to achieve more desirable two- and three-coverage rates with a slight decrease (<3%) in the one-coverage rate.

**Figure 6:**
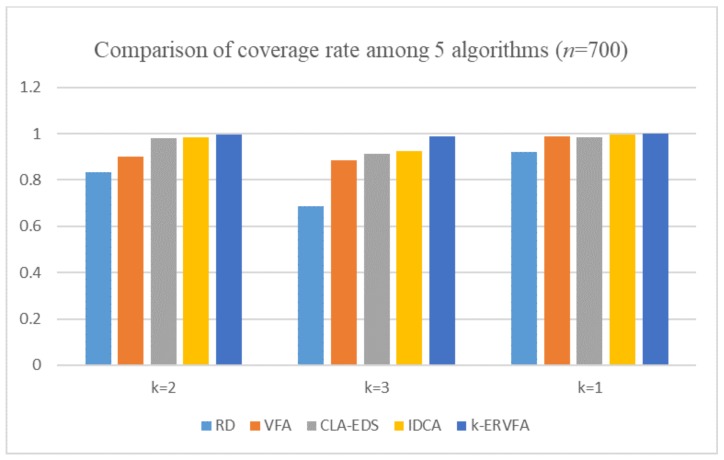
Comparison of the coverage rate among the five algorithms (*n* = 700).

In addition, in order to prove that it is reasonable to deploy 650 nodes in practical applications, we added a set of experiments with 700 nodes, and the experimental results are shown in Figure 6. Deploying 700 sensor nodes could achieve a 100% one-coverage rate and at least an 89% two- and three-coverage rate. However, compared with the result of 650 nodes, the effect of the improvement was not obvious. It also caused the redundancy of nodes and the increase of the deployment cost. In fact, with dynamic adjustment of node position based on the *k*-resultant force, *k*-ERVFA achieved a satisfactory two- and three-coverage rate (though the one-coverage rate decreased slightly) using only 650 nodes. Therefore, it is reasonable and optimal to deploy 650 nodes in the real scenario.

**Figure 7:**
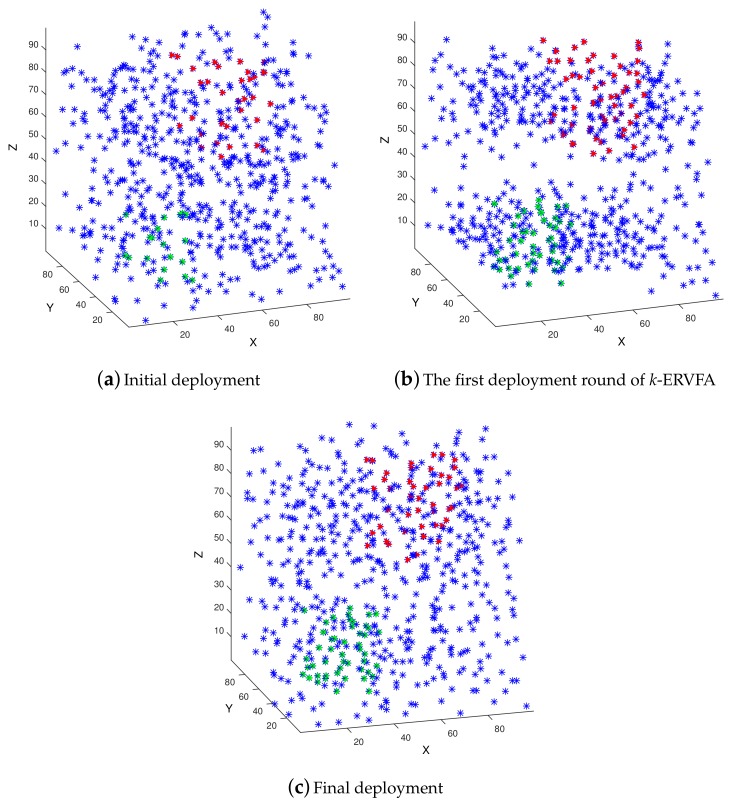
The deployment of 600 sensors in the 3D underwater area. (**a**) Initial deployment; (**b**) The first deployment round of k-ERVFA; (**c**) Final deployment.

Figure 7a–c demonstrate the deployment of 600 sensor nodes in the 3D underwater area with the parameters shown in Table 5. Figure 7a shows the initial Random Deployment (RD) where the green dots are the sensor nodes located in three-coverage requirement sub-region $A_{3,1}$, the red triangles are the nodes in two-coverage requirement sub-region $A_{2,1}$, and all other blue asterisks are sensors in one-coverage requirement sub-regions. It can be seen that sensor nodes were non-uniformly distributed with RD, and the number of nodes in $A_{3,1}$ and $A_{2,1}$ were few. Figure 7b shows the distribution of nodes after the first round of *k*-ERVFA, i.e., the round in which spheres with three-equivalent radius ($r_{3}=r/3$) were applied to achieve high a three-coverage rate. Due to the *k*-attraction of sub-region $A_{2,1}$, nodes would aggregate towards $A_{2,1}$, and the two-coverage rate increased. However, the node distribution in $A_{1,1}$ was still non-uniform, which lead to many coverage breaches. Figure 7c is the ultimate distribution after *k*-ERVFA has been performed. Compared with Figure 7b, nodes were more uniformly distributed in $A_{3,1}$ and $A_{2,1}$ due to the effect of the fix and even algorithm. There were fewer coverage breaches in $A_{1,1}$ so the one-coverage rate increased accordingly. Eventually, the *k*-coverage rates achieved by *k*-ERVFA with 600 nodes were: 92.67% for one-coverage, 97.54% for two-coverage, and 95.22% for three-coverage, which was consistent with the theoretical analysis.

## 6. Further Discussions

### 6.1. The Principle of the Fix and Even Algorithm

After completing Step 1 of the *k*-ERVFA algorithm, the redeployment process in the ${k_{i}}^{\prime }$-coverage requirement sub-regions was only partially done. For now, we know that either the coverage rate in all ${k_{i}}^{\prime }$-coverage requirement sub-regions reached η or the iteration number reached $N_{max}$. The latter is the more common case in practice. It is worth emphasizing that the value of $N_{max}$ should not be too large due to restrictions of energy and running time. Namely, in most cases, the iteration number reached $N_{max}$, but the ${k_{i}}^{\prime }$-coverage rate inside $A_{{k_{i}}^{\prime },j}$ was far from satisfaction.

However, if we continue the node redeployment process in Step 2 of *k*-ERVFA, some nodes already inside $A_{{k_{i}}^{\prime },j}$ may move away from $A_{{k_{i}}^{\prime },j}$ due to the attraction from other sub-regions, causing unnecessary moving and computing costs. In order to avoid this situation, here we consider site preservation and bound these nodes inside $A_{{k_{i}}^{\prime },j}$, i.e., these nodes cannot move out of $A_{{k_{i}}^{\prime },j}$ and will not participate in future redeployment rounds. This is the operation of “fix”.

Now that the nodes are fixed inside $A_{{k_{i}}^{\prime },j}$, their distribution is still uneven, and the ${k_{i}}^{\prime }$-coverage rate is not satisfactory. Naturally, we want to homogenize the node distribution inside $A_{{k_{i}}^{\prime },j}$. This is the operation of “even”.

With the fix and even algorithm, we fixed the sensor nodes inside the sub-region and then homogenized the node distribution, for the purpose of achieving a higher ${k_{i}}^{\prime }$-coverage rate inside $A_{{k_{i}}^{\prime },j}$.

### 6.2. Discussion of the Value of θ(k,η)

In this section, we discuss whether there is a rough acceptable range for the value of θ(k,η) to guide node deployment in practice.

As shown in Figure 3, when θ<1.4, some individual points in the curve of the f-coverage rate were above the corresponding points in the curve of the three-coverage rate, and these two curves tended to merge when θ>2.2. For a given θ, θ·k increased with *k*, which provided more extra nodes. Namely, if we used the same θ(k,η) for different *k* values, then the redundancy of nodes in cases with high *k* values was excessive and would result in extra deployment cost. Therefore, we conjectured that with the increment of *k*, the optimal θ(k,η) was no more than O(k) or even had an upper bound of a constant.

**Conjecture** **1.** *1<θ(k,η)≤2.3 when η is 89% and k≥2.*

**Proof.**  It is difficult to give the strict mathematical proof of the conjecture, so we discuss the problem by performing statistical simulations.

**Figure 8:**
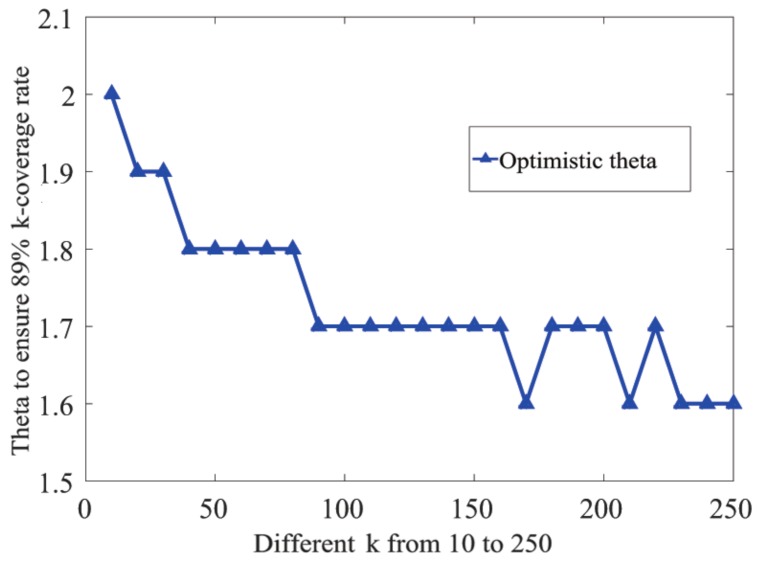
Different *k* and the corresponding optimal θ.

Let η be 89%. We ran the OθSA (Optimal θ Search Algorithm) simulation experiments with *k* values varying from 1 to 300. We had the maximum of θ(k,η) as $\max{\left\{\theta \left(k,89\%\right)\right\}}_{k=1∼300}$ = 2.3 when *k* = 5. Namely, for k=1∼300, the value of optimal θ(k,η) had a numerical upper bound of 2.3. The simulation result shown in Figure 8 also suggests that θ(k,η) decreased when *k* increased. In general, the value of θ(k,η) decreased when *k* increased from 10 to 250.

For higher *k* values, the node deployment interval ($l=2r/(m\cdot \sqrt[{3}]{k}$)) was rather small, which led to a high time complexity of OθSA. Therefore, we only carried out the OθSA for several individual *k* values to testify to our hypothesis. We obtained θ(500, 89%) = 1.6 when *k* = 500, θ(700, 89%) = 1.6 when *k* = 700, and θ(900, 89%) = 1.6 when *k* = 900. None of the above θ values was larger than 2.3, which was consistent with our hypothesis. Note that θ(k,η) seemed to approximate to some lower bound when *k* was large, and this lower bound should be greater than one.

When θ was set to be one, we performed simulations with different *k* values and calculated the *k*-coverage rate. We obtained a 28.79% *k*-coverage rate when *k* = 500, a 29.96% *k*-coverage rate when *k* = 700, and a 26.11% when *k* = 900. The average *k*-coverage rate obtained was 33.32%. This suggests that θ(k,η) should still be greater than one even when *k* is very large. In fact, when we increased θ to 1.1, the average *k*-coverage rate was then 61.71%, which almost doubled the *k*-coverage rate when θ=1. This shows that the redundancy caused by θ plays a crucial role in the improvement of *k*-coverage. Even a slight increment of θ(k,η) over one could significantly increase the *k*-coverage rate.

In summary, we had 1<θ(k,η)≤2.3 when k≥2. □

The value of θ(k,η) was within a small constant range and had an upper bound, independent of *k*. It can be seen that the actual deployment volume density ensured the coverage was linearly related to *k*, and the deployment cost was acceptable even if the value of *k* was large.

### 6.3. Discussions of the Value of r, $N_{max}$, and $\Delta L_{max}$

#### 6.3.1. Discussion of the Value of *r*

To study the influence of the value of *r* on the performance of *k*-ERVFA, we conducted simulation experiments with different *r* values. Most of the simulation parameters were the same as those in Table 5, except for the coverage radius *r* and the number of sensor nodes.

**Figure 9:**
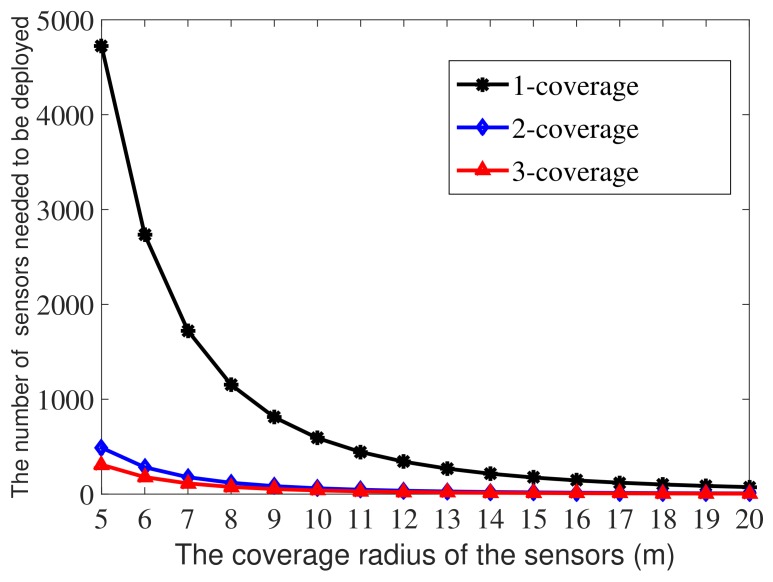
The correlation between *r* and the number of nodes needed to achieve an 89% *k*-coverage rate.

According to Equation (14), the total number of sensor nodes needed to obtain a satisfactory *k*-coverage rate (89% in this paper) is proportional to $1/r^{3}$. Figure 9 shows the minimum number of nodes needed in each sub-region to meet the corresponding *k*-coverage (*k* = 1, 2, 3) requirement with *r* varying from 5 m to 20 m.

As shown in Figure 9, with the increase of the node coverage radius *r*, the number of required nodes decreased rapidly at first and then became stable. This was because when the coverage radius was small, the coverage area was very limited, and a large number of nodes were needed to achieve the required coverage rate. As *r* increased, the coverage rate increased, and the number of nodes required decreased. When *r* reached a certain threshold, the number of required nodes tended to stabilize due to comprehensive coverage and network connectivity.

In addition, the coverage radius of the node was determined by its power. The increase of power would increase the energy consumption of the system and reduce the network life, so it was necessary to consider comprehensively the coverage and energy consumption of the system to choose the appropriate node radius in the practical applications.

In the case of *r* = 1 m, the calculated number of sensor nodes was: $5.9041*10^{5}$ in the one-coverage requirement sub-region, 61,115 in the two-coverage requirement sub-region, and 38,675 in the three-coverage requirement sub-region. Note that the total number of sensor nodes needed when *r* = 10 m was 692 (seen in Section 5), and it is obvious that with different coverage radius, the number of sensor nodes needed differed greatly. Fewer sensors were needed when the coverage radius was large, which is in consistent with practical experience.

**Figure 10:**
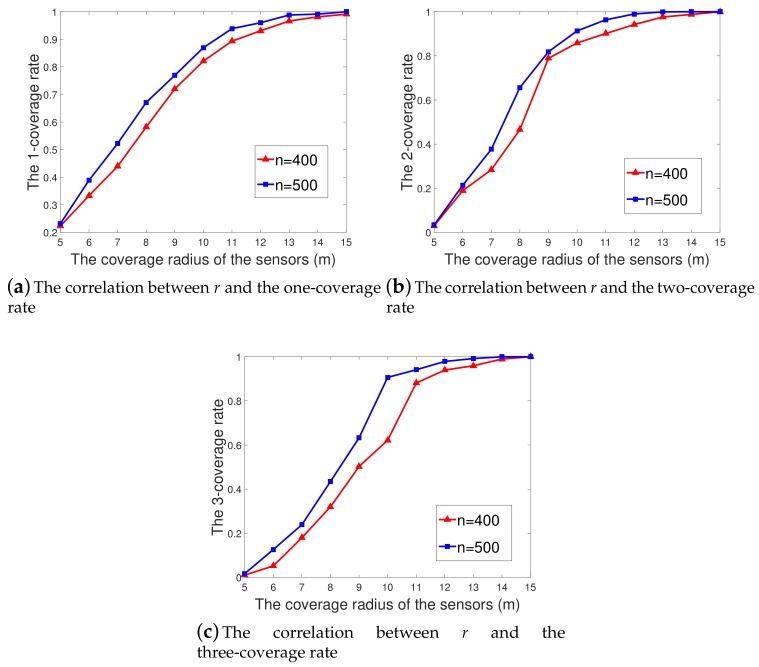
The correlation between *r* and the coverage rate. (**a**) The correlation between *r* and the one-coverage rate; (**b**) The correlation between *r* and the two-coverage rate; (**c**) The correlation between *r* and the three-coverage rate.

Figure 10a–c show the curves of the *k*-coverage rate (*k* = 1, 2, 3) for different sensor numbers (400 and 500) when *r* varied from 5 m to 15 m.

It should be noted that when *r* was small (e.g., r=5 m), the calculated total number of sensor nodes needed to obtain a satisfactory *k*-coverage rate was quite large (more than 5000), while we set *n* as 400 or 500 in the simulations. This is the problem of node deployment in sparse sensor networks. Because *k*-ERVFA adopts a greedy strategy that considers node deployment in high *k*-coverage requirement sub-regions as the first priority, the coverage rate in the low *k*-coverage requirement sub-regions achieved in sparse sensor networks was rather poor. Therefore, the A*k*-ERVFA (seen in Section 6.4.1) was applied here to improve the performance in sparse sensor networks, especially for sub-regions with a low *k*-coverage requirement.

For higher *r* values (r≥9 m), we tended to use the original *k*-ERVFA algorithm to highlight its advantage of improving the *k*-coverage rate with high *k* values.

As shown from Figure 10a–c, the *k*-coverage rate in the corresponding area increased with the value of *r*, which is consistent with practical experience. When r=5 m and n=500, which is the aforementioned case of a sparse sensor network, the initial 1-, 2-, and 3-coverage rates in the corresponding sub-regions were 19.06%, 0.96%, and 0.37%. These coverage rates increased to 23.17%, 3.34%, and 1.68% in the simulation experiments of A*k*-ERVFA.

The two-coverage rate and three-coverage rate increased significantly when 5 m ≤r≤ 10 m, as shown in Figure 10b,c. This increase tendency slowed down a bit when *r* was greater than 10 m. In particular, when r=12 m and n=500, the initial 1-, 2-, and 3-coverage rates were 94.36%, 89.7%, and 57.13% and were improved to 96.02%, 98.99%, and 97.89% by *k*-ERVFA. These *k*-coverage rates were rather impressive compared with the *k*-coverage rate obtained in Section 5 with 650 sensor nodes, considering only 500 sensors were deployed here. This indicates that a slight increment of *r* can lead to a significant improvement of the *k*-coverage rate. When r=15 m and n=500, the initial 1-, 2-, and 3-coverage rates were 99.13%, 99.94%, and 97.51%, and they all increased to 100% after redeployment with *k*-ERVFA. In conclusion, the value of *r* had a significant influence on the variation of the *k*-coverage rate.

Obviously, the *k*-coverage rate achieved by *k*-ERVFA was 100% for higher *r* values (r≥15 m). Therefore, for the diverse 3D underwater *k*-coverage requirement scenarios described in this paper, 400∼500 sensor nodes with a physical coverage radius of 10 m∼15 m can guarantee a satisfactory *k*-coverage rate.

#### 6.3.2. Discussion of the Value of $N_{max}$

**Figure 11:**
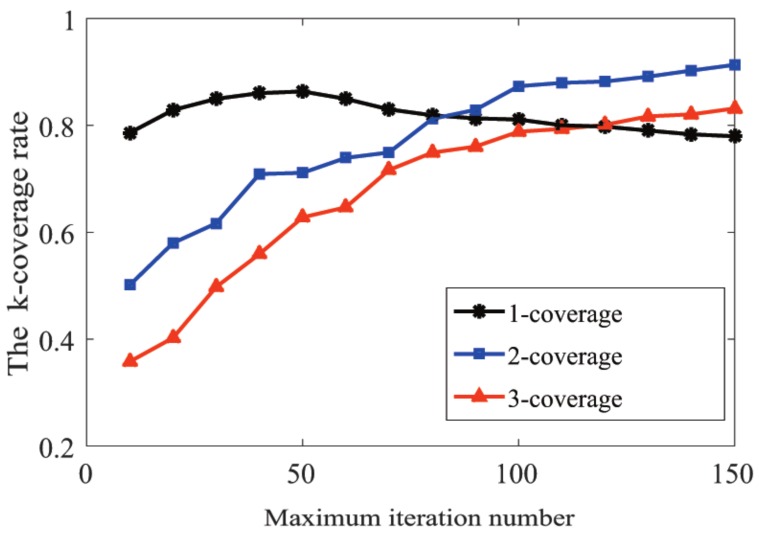
The correlation between $N_{max}$ and the *k*-coverage rate.

In this section, we study the influence of the maximum iteration number on the performance of the *k*-ERVFA algorithm. Similarly, the setting of the underwater monitoring area and all simulation parameters except for $N_{max}$ were the same as those in Section 5. We carried out simulations with *n* = 400 and $N_{max}$ increasing from 10–150 by steps of 10. Figure 11 shows the different *k*-coverage rates obtained by *k*-ERVFA with different values of $N_{max}$. It should be mentioned that in order to eliminate the difference from the initial coverage rate caused by the initial random deployment, the initial sensor distributions in different rounds were set to be identical. The initial 1-, 2-, and 3-coverage rates were 77.51%, 41.96%, and 24.81%, respectively.

Besides, we modified Step 2 of the original *k*-ERVFA so that the loop ends only if the iteration number has reached $N_{max}$, i.e., the judging condition of whether the coverage rate has reached η was removed. The “fix” operation was also abolished; thus, sensor nodes moved more freely in order to demonstrate the effect of $N_{max}$ and the greedy strategy of *k*-ERVFA.

As expected, the two-coverage rate and three-coverage rate increased significantly with the growth of $N_{max}$, as shown in Figure 11. When $N_{max}$ = 150, the two-coverage rate obtained was 91.33%, and the three-coverage rate was 83.17%. They increased by 49.37% and 58.36%, respectively, compared to the initial coverage rate.

The one-coverage rate increased smoothly when $N_{max}$ increased from 10 to 50, reaching a maximum coverage rate of 86.35% when $N_{max}$ = 50. However, it decreased gradually when $N_{max}$ was greater than 50 and reached a minimum of 77.96% when $N_{max}$ = 150. It is reasonable to predict that the two- and three-coverage rate will keep growing, while the one-coverage will decrease when $N_{max}$ keeps increasing. In fact, when $N_{max}$ was small, the homogenization effect from repulsion was the dominant factor to facilitate the increase of the one-coverage rate. When $N_{max}$ was large, the *k*-attraction of the sub-regions with the high *k*-coverage requirement was dominant, and more sensors moved into high *k*-coverage sub-regions, which led to the increment of the high *k*-coverage rate and the decline in the one-coverage rate. Larger $N_{max}$ also highlighted the characteristics of the greedy strategy adopted by the *k*-ERVFA algorithm, as mentioned above.

According to Figure 11, the two-coverage and three-coverage rate increased significantly when $N_{max}$ grew from 40 to 100, and this increasing tendency slowed down when $N_{max}$ was over 100. When $N_{max}$ was around 100, the one-coverage rate was also acceptable. Considering that the excessively large value of $N_{max}$ would result in unnecessary running time and energy consumption, it was appropriate to set $N_{max}$ as 80 to 100 in the underwater monitoring scenario described in this paper. In the practical scenario, an appropriate value of $N_{max}$ is important to achieve the better trade-off between coverage requirements and network cost.

#### 6.3.3. Discussion of the Value of $\Delta L_{max}$

In this section, we study the effect of $\Delta L_{max}$, which is the maximum possible moving distance (along the *Z*-axis) of the sensor node in each iteration. Similarly, the parameters except for $\Delta L_{max}$ were identical to those in Section 5. We carried out simulations with *n* = 400 and fixed the initial distribution of sensor nodes throughout the simulation process for the same reason mentioned in Section 6.3.2. The initial 1-, 2-, and 3-coverage rates were 77.51%, 41.96%, and 24.81%, respectively.

**Figure 12:**
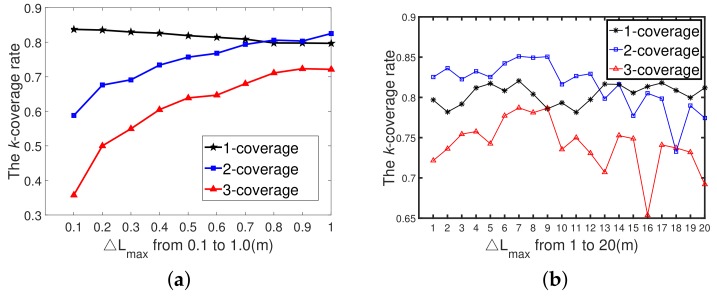
The correlation between $\Delta L_{max}$ and the *k*-coverage rate. (**a**) The correlation between $\Delta L_{max}$ and the *k*-coverage rate (*n* = 400, *r* = 10 m, $\Delta L_{max}=0.1∼1$ m); (**b**) The correlation between $\Delta L_{max}$ and *k*-coverage rate (*n* = 400, *r*=10 m, $\Delta L_{max}=1∼20$ m).

Figure 12a shows the different *k*-coverage rates achieved by the *k*-ERVFA with $\Delta L_{max}$ growing from 0.1 m to 1 m with a step size of 0.1 m. When $\Delta L_{max}$ = 0.1 m, the final 1-, 2-, and 3-coverage rates were 83.72%, 58.78%, and 35.76%, respectively. Compared with the initial *k*-coverage rates, the improvement by *k*-ERVFA was inappreciable. The reason was that compared to the size of the underwater monitoring area (100 m ∗ 100 m ∗ 100 m), $\Delta L_{max}$ was so small (0.1m) that sensor nodes could not reach the desirable destinations based on *k*-ERVFA within the maximum iteration times ($N_{max}$ = 100); thus, the *k*-coverage rate obtained was unsatisfactory. However, the effect of *k*-ERVFA improved with the increasing of $\Delta L_{max}$. The decline in the one-coverage rate shown in Figure 12a was due to the fact that more sensor nodes moved into the two- and three-coverage requirement sub-regions when $\Delta L_{max}$ increased.

In Figure 12b, when $\Delta L_{max}$ grew from 1 m to 20 m with a step size of 1 m, all the *k*-coverage rates oscillated with the growth of $\Delta L_{max}$. When the value of $\Delta L_{max}$ was small ($1m\leq \Delta L_{max}\leq 7$ m), the moving range of sensor nodes in each iteration was longer compared with those in Figure 12a ($\Delta L_{max}<1$ m). With the same iteration number, the effect of the redeployment process was more significant; thus, all the *k*-coverage rates increased. However, when $\Delta L_{max}$ kept increasing ($\Delta L_{max}>7$ m), the moving range of sensor nodes in each iteration was so long that their motion pattern turned into a certain kind of disordered oscillation. Hence, the *k*-coverage rates obtained by *k*-ERVFA could not satisfy the actual requirements.

In conclusion, the value of $\Delta L_{max}$ should be moderate. If $\Delta L_{max}$ is too small (≤1 m), then sensors cannot reach their final destinations before the redeployment process is terminated. However, the excessively large value of $\Delta L_{max}$ (≥10 m) will cause nodes to drift and oscillate; thus, the performance of *k*-ERVFA will be unpredictable. We can see from Figure 12b that when $\Delta L_{max}$ = 7 m, the 1-, 2-, and 3-coverage rates all reached their maximum value. Therefore, $\Delta L_{max}$ = 7 m was the optimal value under such simulation conditions ($n=400,N_{max}=100,r=10$ m).

Table 6 shows the optimal $\Delta L_{max}$ for different total numbers of sensors (when *r* = 10 m). We can see that with the node number increasing, the optimal value of $\Delta L_{max}$ decreased gradually. The reason was that with more sensor nodes deployed in the area, the average distance between the coverage breached, and the neighboring sensor nodes decreased; thus, the appropriate moving distance of the sensor node in each iteration (which was decided by $\Delta L_{max}$ in Equation (22)) decreased as well. Therefore, the key to application implementation lies in how to obtain the optimal value of $\Delta L_{max}$ according to the total number of nodes in the actual scenario.

### 6.4. The Improvement of k-ERVFA

The underwater sensor network is closely related to many marine environment factors, such as fish stock interference and tidal disturbances. For example, when the fish school destroy the nodes or the nodes and equipment age and fail, the previous deployment requirements will be changed to adapt to the new situation. The number of nodes may be insufficient in some scenarios, and even in some extreme conditions, the total number of sensor nodes is so small that the sensor network is quite sparse. In addition, in practical applications, *k*-coverage requirements may change over time according to seasonal changes or actual situations, which is also a real problem that needs to be considered. Aiming at these actual problems mentioned above, we discuss the Ak-ERVFA (Averaged *k*-ERVFA) algorithm for node sparsity and the Ck-ERVFA (Changed *k*-ERVFA) algorithm for time-variant demand, respectively.

#### 6.4.1. A*k*-ERVFA

Due to the strategy taken by *k*-ERVFA, which satisfied the *k*-coverage requirement with high *k* values preferentially and the fact that the total number of nodes was severely limited, the performance of *k*-ERVFA in sparse sensor networks was unsatisfactory. Especially, sub-regions with requirements of low coverage multiplicity tended to be ignored.

We propose the A*k*-ERVFA (Averaged *k*-ERVFA) to improve the performance of *k*-ERVFA in sparse sensor networks, and the modifications are as follows:

1. Let η be 89%; the number of nodes required in different *k*-coverage requirement sub-regions can then be calculated by Equation (14), denoted by $N_{min}\left(r,1,89\%\right)$, $N_{min}\left(r,2,89\%\right)$,…, $N_{min}\left(r,k,89\%\right)$, $N_{min}\left(r,n_{k},89\%\right)$, respectively (*k* = 1, 2, 3, … $n_{k}$).
2. Calculate the proportional factor λ(r,k) for each sub-region $A_{k,j}$ as follows: $\lambda \left(r,k\right)=N_{\min}\left(r,k,0.89\right)/\sum_{i=1}^{n_{k}}N_{\min}\left(r,i,0.89\right)$.
3. Modify the termination condition in Step 2 of *k*-ERVFA; now, the iteration will also be terminated if the number of nodes inside $A_{k,j}$ reaches $\lambda \left(r,k\right)\cdot N_{total}$, where $N_{total}$ is the total number of sensor nodes.

We can see that with A*k*-ERVFA, the number of nodes deployed in any sub-region was proportional to its expected node number calculated by Equation (14). In this way, sub-regions with different *k*-coverage requirements were treated equally to some extent. Thus, the *k*-coverage rate in sub-regions with the requirements of low coverage multiplicity became reasonable.

#### 6.4.2. C*k*-ERVFA

C*k*-ERVFA was defined as the enhanced *k*-ERVFA for time-variant situations. In Section 4, the coverage requirement information of the underwater monitoring area was known in advance and was considered to be fixed throughout the life-cycle of the sensor network. However, the coverage requirement information is often time-variant in practical applications, and we should extend our *k*-ERVFA algorithm so that it may work in such time-variant situations. In fact, this extension is possible when the running time (response time) of *k*-ERVFA is far less than the time interval between changes in coverage requirement information, which is the case in most practical applications.

We propose the enhanced C*k*-ERVFA as follows:

1. Sink nodes collect the latest coverage requirement information, i.e., the geometry attributes and the coverage multiplicities of all sub-regions. Then, the coordinates of the centroids of the sub-regions and the corresponding equivalent radius are calculated. All sub-regions are sorted in descending order of the *k* value. Set *n* as zero.
2. Sink nodes calculate the *k*-resultant force similar to Step 2 in the *k*-ERVFA algorithm.
3. Call the fix and even algorithm similar to Step 3 in the *k*-ERVFA algorithm.
4. Sink nodes send redeployment commands to sensors, and let n=n+1. Check if *n* reaches $k_{n}$, otherwise go to Step 2.
5. Sink nodes monitor the coverage requirement information constantly; go to step 1 if the coverage requirement information changes, otherwise go to Step 5.

In summary, C*k*-ERVFA monitors changes of the coverage requirement information, and node redeployment will be performed according to the latest coverage requirement. In this way, *k*-ERVFA can adapt to underwater monitoring tasks with the time-variant coverage requirement. It should be noted that in C*k*-ERVFA, sink nodes are capable of collecting information about the coverage requirements, as discussed in [28].

## 7. Conclusions and Future Work

In this paper, we studied the diverse *k*-coverage problem in three-dimensional underwater sensor networks. First, we analyzed the minimum density of sensors required to satisfy the different *k*-coverage requirements by dividing the monitoring area into grids. We introduced θ to provide the necessary redundancy of sensor nodes required in practical applications and proposed the OθSA algorithm to decide the appropriate value of θ. Then, we put forward the enhanced virtual force algorithm *k*-ERVFA to solve the diverse *k*-coverage problem. Both theoretical analysis and simulation experiments were carried out to demonstrate the effectiveness and feasibility of our proposed algorithm. Finally, we extended the *k*-ERVFA to A*k*-ERVFA and C*k*-ERVFA so that it could work in sparse sensor networks and time-variant situations.

Moving forward, we plan to combine our approach with methods based on the Voronoi diagram to better discover coverage breaches and further improve the diverse *k*-coverage rate in 3D UWSNs. We also plan to implement the proposed algorithm with a testbed to evaluate the performance of *k*-ERVFA in real-world application scenarios while taking the influence of marine environment factors and measurement error into account.

## Figures and Tables

**Table 1:** Deployment classification of UWSN.

Scheme	Sea-Column Deployment		Sea-Bottom Deployment
Location	3D Underwater Space		The Bottom of the Sea
Distribution Mode	Uniform deployment	Non-uniform deployment	Nodes are deployed in the intersections of the grids
	Distributed uniformly in the monitoring area	Deployed non-uniformly according to the distribution states of underwater targets	
Characteristics	It cannot meet actual demand	A well-designed protocol and a deployment algorithm are required	The 3D properties of underwater space are not taken into account

**Table 2:** Description of the main notations. ERVFA, Equivalent Radius enhanced Virtual Force Algorithm.

Notation	Description
*r*	The coverage radius of sensor nodes
*k*	The multiplicity of the coverage requirement
$r_{k}$	The *k*-equivalent radius
$A_{k,i}$	The *i*th sub-region with the *k*-coverage requirement
$V_{A_{k,i}}$	The volume of $A_{k,i}$
$p_{k,i}$	Some point inside $A_{k,i}$
$C_{k,i}$	The *k*-coverage rate of $A_{k,i}$
$S_{x}$	The *x*th sensor node
$L_{0}$	The side length of the underwater monitoring area
η	The satisfactory coverage rate
θ	The node redundancy coefficient
$\rho_{min}$	The minimum deployment density of sensor nodes
$n_{min}$	The minimal number of sensor nodes
$K_{conf}$, $K_{attr,k}$, $K_{Ob,k}$	The virtual force coefficients
$\Delta L_{max}$	The maximum moving distance of sensor nodes in each iteration
$N_{max}$	The maximum iteration times in each round of *k*-ERVFA

**Table 3:** The η value and the matching θ value in different cases.

		*k*	2	3	4	5
	θ					
η						
88%			1.9	1.9	1.9	2.2
89%			2.0	2.0	2.0	2.3
90%			2.1	2.2	2.1	2.4

**Table 4:** The minimum *m* and corresponding volume density to ensure an 89% *k*-coverage.

*k*	1	2	3	4	5	…
θ(k,η)	1	2	2	2	2.3	…
$m_{\min}\left(k,\eta \right)$	$\sqrt{3}$	$\sqrt[{3}]{\frac{12}{\pi }}$	$\sqrt[{3}]{\frac{12}{\pi }}$	$\sqrt[{3}]{\frac{12}{\pi }}$	$\sqrt[{3}]{\frac{13.8}{\pi }}$	...
$\rho_{\min}\left(r,k,\eta \right)$	$\frac{3\sqrt{3}}{8r^{3}}$	$\frac{3}{\pi r^{3}}$	$\frac{9}{2\pi r^{3}}$	$\frac{6}{\pi r^{3}}$	$\frac{8.625}{\pi r^{3}}$	...

**Table 5:** Simulation parameters. RD, Random Deployment; CLA-EDS, a Cellular Learning Automata-based Enhanced Deployment Strategy; IDCA, Intelligent Deployment and Clustering Algorithm.

Monitoring area	100 m ∗ 100 m ∗ 100 m
$k_{3}$-sub region $A_{3,1}$	$k_{3}$ = 3, $center_{3}$: (25 m, 25 m, 25 m), $size_{3}$ = 30 m ∗ 30 m ∗ 30 m
$k_{2}$-sub region $A_{2,1}$	$k_{2}$ = 2, $center_{2}$: (70 m, 70 m, 70 m), $size_{2}$ = 40 m ∗ 40 m ∗ 40 m
$k_{1}$-sub region $A_{1,1}$	$k_{1}$ = 3, $size_{1}$ = the rest of the monitoring area
Number of sensor nodes	400∼650, with a step size of 50
Coverage radius *r*	10 m
$\Delta L_{max}$	7 m
Contrast algorithms	*k*-ERVFA, RD, VFA, CLA-EDS, and IDCA
Max iteration times $N_{max}$	100
$K_{conf}$, $K_{attr,k}$, $K_{Ob,k}$	$K_{conf}$:$K_{attr,2}$:$K_{attr,3}$:$K_{Ob,2}$:$K_{Ob,3}$ = 1:2:3:2:3

**Table 6:** Optimal $\Delta L_{max}$ for different numbers of nodes.

Number of nodes	400	450	500	550	600	650
Optimal $\Delta L_{max}$(m)	7	7	6	6	6	5

**Figure A1:**
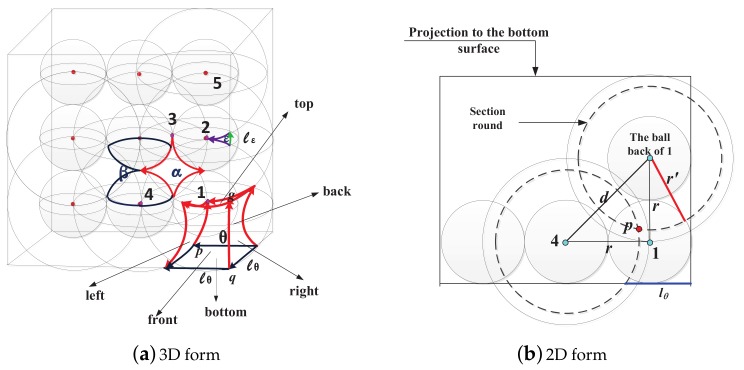
Tangent deployment of two-equivalent radius spheres. (**a**) 3D form; (**b**) 2D form.

**Figure A2:**
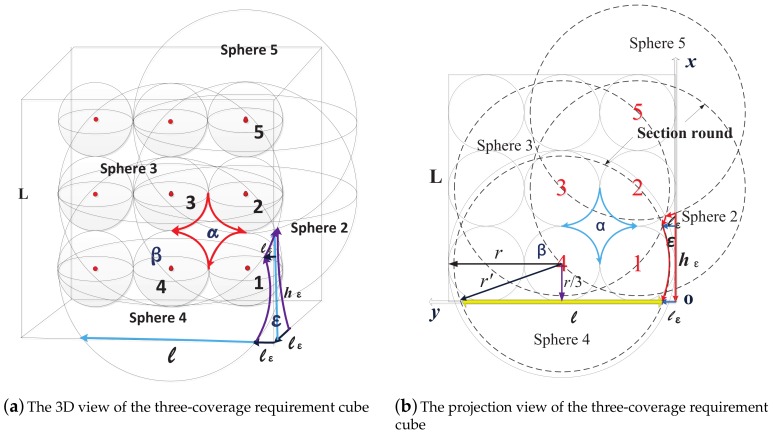
Critical case in the three-coverage requirement region. (**a**) The 3D view of the three-coverage requirement cube; (**b**) The projection view of the three-coverage requirement cube.

**Figure A3:**
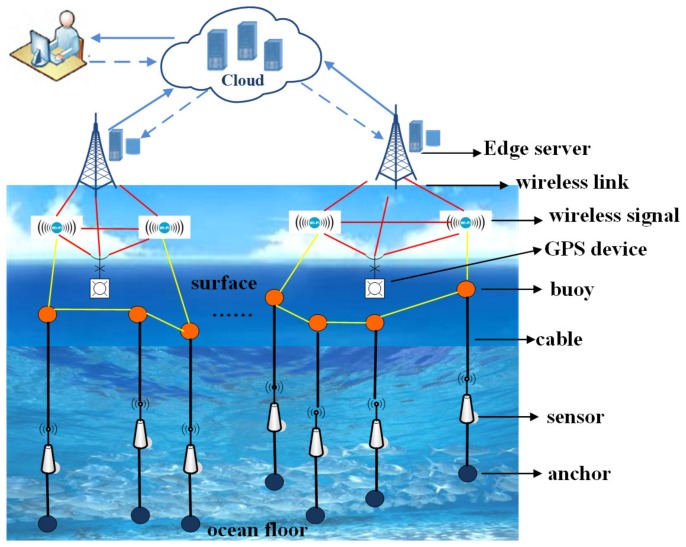
Underwater sensor deployment model.

## Appendix A. Theoretical Analysis of the Effectiveness of the *k*-ERVFA Algorithm

### Appendix A.1. The Effectiveness of k-ERVFA in the Case of k = 2

In this subsection, we perform a theoretical analysis on the correctness and effectiveness of the *k*-ERVFA algorithm when *k* = 2.

According to the *k*-ERVFA algorithm, spheres with a two-equivalent radius ($r_{2}$ = r/2) are deployed in the two-coverage requirement sub-regions. Note that the actual coverage radius is still *r*, as mentioned in Section 3.2.

As shown in Figure A1, the larger spheres are the actual coverage region of the sensor nodes, while the smaller spheres are the two-equivalent radius spheres defined in Definition 6 (seen in Section 3.2). In Figure A1, the two-equivalent radius spheres are tangent to each other, which is considered as the critical case. When adopting the *k*-ERVFA algorithm, neighboring two-equivalent radius spheres tend to overlap with each other, which will result in a higher two-coverage rate than the critical case.

For simplicity, assume that the sub-region is a cube with side length *L* (L≥3r). If *L* is less than 3r, the number of nodes needed can be calculated by Equation (14) in the paper, then these sensor nodes are deployed into the cubic region. In this case, there will be overlapping areas between adjacent two-equivalent spheres, and the actual coverage rate will be greater than the critical case. Based on the discussions above, here we only need to study the critical case in Figure A1 when L≥3r.

There are four kinds of three-dimensional regions that need to be discussed separately. These regions are marked as α,β,ε,andθ in Figure A1, where α∩β∩ε∩θ=Ø.

1. For any point within region α, it is covered by four sensor nodes because it is within the coverage range from Sensor 1 to Sensor 4. Namely, region α is at least four-covered.
2. For any point within region β (β includes the internal part and upper external part of the sphere with the two-equivalent radius of Sensor 4; here, the upper external part is inside the actual coverage sphere of Sensor 4), it is covered by Sensors 3 and 4 at least. Hence, region β is at least two-covered.
3. For any point within region ε (ε is located near the edge in the right front side of the cube), it can only be covered by Sensor 2. Thus, region ε is only one-covered. We need to know the total number of ε regions (denoted by $n_{\varepsilon }$) in the entire cubic in order to calculate the 2-coverage rate. Assume that $L=K\cdot r,K\geq 3,K\in N^{+}$; we derive the total number of region ε as follows (the detailed derivation process can be found in Appendix A.2 for *k* = 3):
  (A1) nε=12·(L2·(rr22)−2)=12(Lr−2)=12(K−2)
4. For any point within region θ (θ is located in the vertices of the cube as shown in Figure A1; θ includes the external part of sphere with the two-equivalent radius of Sensor 1; here, the external part is inside the actual coverage region of Sensor 1), it can only be covered by Sensor 1; thus, region θ is only one-covered. There are eight θ regions in the cube, and each one corresponds to a vertex of the cube.

In order to calculate the two-coverage rate of the entire cubic, we need to estimate the volume of region ε and region θ, which are not two-covered.

The front, bottom, and right surfaces of region θ are flat, while the top, left, and back surfaces are spherical, which are formed by some parts of the spheres with a two-equivalent radius of Sensor 2, 4, and 6.

Based on the symmetry of region θ, we can estimate its volume as follows:

(A2) $$V_{\theta }<l_{\theta }^{3}-\frac{1}{3}S^{\prime }\left(l_{\theta }-\frac{r}{2}\right)\cdot 3=l_{\theta }^{3}-l_{\theta }^{2}\left(l_{\theta }-\frac{r}{2}\right)=\frac{rl_{\theta }^{2}}{2}$$

where ${l_{\theta }}^{3}$ is the volume of the cubic with side length $l_{\theta }$ and $S^{\prime }$ is the area of the square with side length $l_{\theta }$ (these squares share common vertices with the three spherical surfaces of region θ). $\frac{1}{3}S^{\prime }\left(l_{\theta }-\frac{r}{2}\right)$ is the volume of the three pyramids that will be removed to estimate the volume of region θ. These pyramids are based on the aforementioned squares, and their apexes are at the center of the spherical surfaces of region θ. $V_{\theta }$ is less than the value on the right side of Equation (A2) because the spherical surfaces are concave. With knowledge of solid geometry, we have $l_{\theta }^{}=\frac{3-\sqrt{2}}{2}r$. There is a total of eight θ regions in the entire cube. Therefore, the percentage of $V_{\theta }$ in the entire cube is:

(A3) $$\begin{array}{cc} P_{\theta } & =\frac{8V_{\theta }}{V_{cube}}=\frac{4rl_{\theta }^{3}}{L^{3}}<\frac{4rl_{\theta }^{3}}{{\left(Kr\right)}^{3}}\\ & =\frac{{\left(3-\sqrt{2}\right)}^{3}}{2K^{3}}\leq \frac{{\left(3-\sqrt{2}\right)}^{3}}{2\cdot 3^{3}}=0.0738 \end{array}$$

Similarly, we can estimate the volume of the region ε (denoted by $V_{\varepsilon }$) as follows:

(A4) $$\begin{array}{cc} V_{\varepsilon } & <l_{\theta }^{2}l_{\varepsilon }-\left(2\cdot \frac{1}{3}S_{1}\frac{l_{\varepsilon }}{2}+\frac{1}{3}S_{2}\left(l_{\theta }-\frac{r}{2}\right)+\frac{1}{3}S_{3}\left(l_{\theta }-\frac{r}{2}\right)\right)\\ & =\frac{1}{3}l_{\theta }^{}l_{\varepsilon }r=\frac{1}{3}\cdot \frac{3-\sqrt{2}}{2}\left(2-\sqrt{2}\right)r^{3}=0.1548r^{3} \end{array}$$

where $l_{\theta }^{2}l_{\varepsilon }$ is the volume of the cuboid with height $l_{\varepsilon }$. The terms after the minus sign are the volume of pyramids to be removed. Similarly, $V_{\varepsilon }$ is less than the value on the right side of Equation (A4) due to the concavity of region ε. From Equation (A1), we have obtained the total number of regions ε; therefore, the percentage of $V_{\varepsilon }$ in the entire cube is:

(A5) $$\begin{array}{cc} P_{\varepsilon } & =\frac{n_{\varepsilon }V_{\varepsilon }}{L^{3}}<\frac{12\left(K-2\right)\cdot 0.1548r^{3}}{{\left(K\cdot r\right)}^{3}}\\ & =\frac{1.8579(K-2)}{K^{3}}\leq \frac{1.8579}{3^{3}}=0.0688 \end{array}$$

The two-coverage rate of the entire cube is then:

(A6) $$\begin{array}{cc} P_{reality,2} & \geq P_{critical,2}=1-P_{\varepsilon }-P_{\theta }\\ & >1-0.0688-0.0738=0.8574 \end{array}$$

$P_{reality,2}$ is the two-coverage rate obtained by *k*-ERVFA with enough sensor nodes and sufficient running time. $P_{critical,2}$ is the two-coverage rate in the critical case in Figure A1 when spheres with two-equivalent radius are tangent to each other and L=3r.

In Section 5, we set *L* to be 10r in the simulation experiments (*K* = 10). Substituting the *K* value into Equations (A2)–(A6), we have $P_{\theta }<\frac{{\left(3-\sqrt{2}\right)}^{3}}{2\cdot 10^{3}}=0.002$, $P_{\varepsilon }<\frac{1.8579(10-2)}{10^{3}}=0.0149$, and $P_{reality,2}=1-P_{\varepsilon }-P_{\theta }>0.9831$. Namely, a 98.31% two-coverage rate can be achieved theoretically.

Thus far, we have proven that the *k*-ERVFA algorithm achieved over an 85.74% two-coverage rate in and underwater cube with side length L=3r when there were enough sensor nodes and sufficient running time. The two-coverage rate increased with the increase of *L*. Especially, we achieved over a 98.31% two-coverage rate when L=10r.

There is a similar analysis when *k* = 3 is presented in the next subsection (a 96.73% three-coverage rate was achieved). For *k* values greater than four, we propose a different approach to prove that the 100% *k*-coverage rate can be achieved.

When k=1, the *k*-ERVFA algorithm will degenerate to the general virtual force algorithm for 3D UWSNs, for which the effectiveness and correctness have been extensively studied [7,12].

### Appendix A.2. The Effectiveness of k-ERVFA in the Case of k = 3

According to *k*-ERVFA, spheres with a three-equivalent radius ($r_{3}=r/3$) are deployed in the three-coverage requirement sub-regions when k=3. Note that the actual coverage radius was still *r*.

We studied the critical case when spheres with the three-equivalent radius are tangent to each other, as shown in Figure A2. For simplicity, assume that the side length (denoted by *L*) of the entire cubic region is greater than 2r. In fact, when L≤2r, we can calculate the total number of sensors required according to Equation (14) in the paper and deploy these sensors in the cube to guarantee *k*-coverage.

Now we analyze the three-coverage rate of the entire cube in the aforementioned critical case (L>2r). As the physical coverage radius was still *r* and the distance between sensor nodes was 2·r/3, any point in the interior part of the cube was at least four-covered. Therefore, we only need to study the coverage situation in the boundary part, which was near the surfaces and vertices of the cube. There are three kinds of three-dimensional regions that need to be discussed separately. In Figure A2, these regions are marked as α,βandε, where α∩β∩ε=Ø.

1. For any point within region α, similar to the points in the interior part, it can be covered by Sensors 1, 2, 3, and 4, so region α is at least four-covered.
2. For any point within region β (around Sensor Node 4) that is near the boundary surface and not close to the edges of the cube, it is covered by Nodes 1, 3, and 4, so region β is at least three-covered.
3. For any point within region ε that is near the edges of the cube, it can only be covered by Node 1 or both Nodes 1 and 2, so region ε is not three-covered. We need to estimate the volume of region ε and the total number of such regions to calculate the three-coverage rate of the entire cube.

First, we estimated the volume of region ε. Figure A2b is the projection view of Figure A2a on the front surface of the cube. The radius of the circular section of Sphere 4 on the surface was then r′=r2−(r/3)2=22r22r33.

For the chord length *l* in Figure A2a, we have:

(A7) $$l=2\sqrt{r{}^{\prime 2}-{\left(r/3\right)}^{2}}=2\sqrt{{\left(2\sqrt{2}r/3\right)}^{2}-{\left(r/3\right)}^{2}}=2\sqrt{7}r/3$$

The side length of the bottom surface of region ε is:

(A8) $$l_{\varepsilon }=\frac{1}{2}\left(L-l\right)=\frac{1}{2}(L-\frac{2\sqrt{7}r)}{3}$$

We study the boundary situations with L=2r in Figure A2b; thus, we obtain:

(A9) $$l_{\varepsilon }=\left(1-\frac{\sqrt{7}}{3}\right)r=0.1181r$$

Let $h_{\varepsilon }$ be the height of region ε on the edge; we see that the top vertex of region ε is the intersection point of the edge and Section Round 5. With the coordinate system shown in Figure A2b and knowledge of analytical geometry, we can derive the expression regarding Section Round 5 as ${\left(x-\frac{5}{3}r\right)}^{2}+{\left(y-\frac{r}{3}\right)}^{2}={\left(\frac{2\sqrt{2}}{3}r\right)}^{2}$. Let *y* be zero, and $h_{\varepsilon }$ is calculated as:

(A10) $$h_{\varepsilon }=\left(\frac{5-\sqrt{7}}{3}\right)r=0.7847r$$

Region ε is a concave polyhedron (similar to $V_{\theta }$ in Figure A1) with concave surfaces, so its volume $V_{\varepsilon }$ is less than the volume of the corresponding cuboid, which has the bottom area of ${l_{\varepsilon }}^{2}$ and height $h_{\varepsilon }$.

(A11) $$V_{\varepsilon }<{l_{\varepsilon }}^{2}h_{\varepsilon }={\left(1-\frac{\sqrt{7}}{3}\right)}^{2}\left(\frac{5-\sqrt{7}}{3}\right)r^{3}=0.>0109r^{3}$$

Now, we consider the total number of regions like region ε. Note that region ε appears on the edges of the cube repeatedly with an interval length of *r*. The cube has 12 edges, so there are 12L/r of such regions in total.

Therefore, the total volume of ε regions is estimated as:

(A12) $$V_{\varepsilon -total}<<12\cdot \frac{L}{r}V_{\varepsilon }<0.1308Lr^{2}$$

Now, we consider the entire cubic region and set its side length as $L=M\cdot 2r/3,M\geq 3,M\in N^{+}$, so the percentage of the total volume of all ε regions in the entire cubic region is:

(A13) $$\begin{array}{cc} P_{\varepsilon } & =\frac{V_{\varepsilon -total}}{V_{cube}}<<\frac{0.1308Lr^{2}}{L^{3}}=\frac{0.1308r^{2}}{{\left(M\cdot \frac{2}{3}r\right)}^{2}}\\ & =\frac{0.2943}{M^{2}}\leq \frac{0.2943}{3^{2}}=0.0327 \end{array}$$

So far, our discussion has been based on the critical situation where spheres with *k*-equivalent radius are tangent to each other. The three-coverage rate of the critical case can be denoted by $P_{critical,3}$. In practice, with enough sensors and sufficient running time, the spheres with *k*-equivalent radius will overlap with each other so the three-coverage rate achieved by *k*-ERVFA (denoted as $P_{reality,3}$) will be better than $P_{critical,3}$, i.e.,

(A14) $$P_{reality,\;3}\geq P_{critical,\;3}=1-P_{\varepsilon }>>1-0.0327=0.9673$$

It can be found that in Equation (A13), $P_{\varepsilon }$ decreases when *L* increases. Therefore, the three-coverage rate in larger underwater areas will be greater. For example, when M=10 (L=20r/3), we have $P_{\varepsilon }\leq 0.0029$ and $P_{reality,3}\geq 0.9971$.

### Appendix A.3. Discussion of the Effectiveness of the k-ERVFA Algorithm When k ≥ 4

In the case of k≥4 ($k\in N^{+}$), suppose that sensor nodes are uniformly deployed in a 3D underwater cube with side length *L*. Without loss of generality, let *L* be the integral multiple of $2r_{k}$, i.e., $L\;\;\mathrm{mod}\;\;(2r_{k})=0$, where $r_{k}=r/k$ is the *k*-equivalent radius. We study the critical case where spheres with the *k*-equivalent radius are tangent to each other, as shown in Figure A1 and Figure A2. Therefore, the total number of sensor nodes in the cube is $(L/2r_{k}{)}^{3}=L^{3}/8r_{k}^{3}=L^{3}/8{\left(r/k\right)}^{3}=k^{3}L^{3}/8r^{3}$, and the average volume density of node deployment is $\rho =\frac{k^{3}L^{3}}{8r^{3}}/L^{3}=\frac{k^{3}}{8r^{3}}$.

For some point *p* in the cubic region, it is covered by sensor $S_{i}$ only if the Euclidean distance between *p* and $S_{i}$ is less than the coverage radius of $S_{i}$, i.e., $d_{pS_{i}}\leq r$. We define the under-covered region of point *p* (denoted by $R_{p}$) as follows:

1. For any point in $R_{p}$, its Euclidean distance to point *p* should be less than *r*;
2. The density of node deployment ρ in $R_{p}$ should be a non-zero constant.

Point *p* is *k*-covered if there are more than *k* sensor nodes in $R_{p}$, i.e., $\rho \cdot V_{R_{p}}\geq k$. Based on this definition, we consider the *k*-coverage rate in the aforementioned cubic region. The problem can be divided into two different cases:

1. For any point *p* in the interior part of the cubic region, $R_{p}$ is a sphere center at *p* with the radius of *r*. The average number of sensor nodes in $R_{p}$ is then: $\frac{4}{3}\pi r^{3}\cdot \rho =\frac{4}{3}\pi r^{3}\cdot \frac{k^{3}}{8r^{3}}=\frac{\pi k^{3}}{6}$, so point *p* is $\frac{\pi k^{3}}{6}$-covered. Let $f\left(k\right)=\frac{\pi k^{3}}{6}-k$; we have its derivative as $f^{\prime }\left(k\right)=\frac{\pi }{2}k^{2}-1>0$ when k>1. Note that f(2)=4π/3−2>0, and we have f(k)>0($\frac{\pi k^{3}}{6}>k$) for ∀k≥2; thus, point *p* is at least *k*-covered. Therefore, in the critical case where spheres with *k*-equivalent radius are tangent to each other, the *k*-coverage rate of the interior part of the cubic region is 100% when k≥2. However, the discussion above is based on the average density, and the coverage rate in some sub-regions may not reach 100% in practice due to the non-uniform distribution of sensor nodes.
2. For any point *p* that is near the boundary surfaces and vertices of the cubic region, according to the definition, $R_{p}$ is no longer a complete sphere in this case. Here, the sphere is cut by the surface of the cube, and there are no sensors in the part outside the cube. Therefore, the volume of $R_{p}$ is now $m\cdot \frac{4}{3}\pi r^{3}$ with 1/8≤m≤1. In particular, m=1/8 is the case when *p* coincides with the eight vertices of the cube. The average number of sensor nodes in $R_{p}$ is then $m\cdot \frac{4}{3}\pi r^{3}\cdot \rho =\frac{4m\pi r^{3}}{3}\cdot \frac{k^{3}}{8r^{3}}=\frac{m\pi k^{3}}{6}$. Similarly, let $f\left(k\right)=\frac{m\pi k^{3}}{6}-k$, and we get $f^{\prime }\left(k\right)=\frac{\pi m}{2}k^{2}-1$, 1/8≤m<1. Let *m* be 1/8, which corresponds to the minimum of f(k) and $f^{\prime }\left(k\right)$, and we have f(4)=0.1888>0 and $f^{\prime }\left(4\right)=\pi -1>0$. As f(k) and $f^{\prime }\left(k\right)$ increase with *k*, it can be proven that f(k)>0($\frac{m\pi k^{3}}{6}>k$), when k≥4 and m=1/8. Namely, point *p* can be *k*-covered even if it coincides with the eight vertices of the cubic region. For other cases with various *m* values (1/8<m<1), the volume of $R_{p}$ is greater than the case with m=1/8, which means there are more sensor nodes in $R_{p}$. Therefore, point *p* is also at least *k*-covered.

Based on the discussions above, points in the interior part of the cubic region are *k*-covered when k≥2, and points that are near the boundary surfaces and vertices of the cubic region are *k*-covered when k≥4. Theoretically, the *k*-coverage rate of the cubic region will reach 100% when k≥4.

In fact, for the case of k≥4, the same result can be obtained with the approach given in Appendix A.2.

In conclusion, with enough sensor nodes and sufficient running time, the *k*-ERVFA algorithm will achieve the following *k*-coverage rates in the critical case where spheres with *k*-equivalent radius are tangent to each other:

- Over an 85.74% two-coverage rate for the two-coverage requirement sub-regions;
- Over a 96.73% three-coverage rate for the three-coverage requirement sub-regions;
- A 100% *k*-coverage rate for the *k*-coverage requirement sub-regions when $k\geq 4,k\in N^{+}$.

## Appendix B. Selection of the Underwater Deployment Model

The deployment model we adopted in this paper is the 3D underwater sensor deployment model proposed in [45]. We also adopted the system proposed in [46], which can adjust the depth of sensors automatically. In general, a cloud-based architecture was proposed to provide a high quality of service for UWSNs. However, UWSNs also require low latency, and the central servers need to store and transmit tremendous data. Edge computing can overcome these drawbacks of traditional cloud computing. Therefore, we adopted the edge computing architecture, which performs the related tasks of UWSNs at the nearby edges of networks. The details of our model are shown in Figure A3. Initially, all sensor nodes were scattered into the target sea area from aircraft or ships, and they were then fixed by anchors on the seafloor. Rigid cables were used to prevent buoys from drifting, which would cause sensors to deviate from the target sea area. The length of the cable was adjustable; thus, the depth of the sensor node could be adjusted.

As for the transmission medium, the models proposed by [37,47,48] used an underwater acoustic link. However, due to the limitations of current technology, the underwater acoustic link suffers from issues like low bandwidth, high latency, path loss, noise [49,50], multi-path, etc. Here, we selected the wired cable as the transmission medium between underwater sensor nodes and buoy nodes. Buoy nodes were equipped with wireless communication modules, which received data collected by sensor nodes and transmitted the data to the edge servers. Edge servers became responsible for a subset of tasks in their close vicinity. The buoy nodes also acted as relays for communications between sensor nodes and were equipped with a positioning module (such as a GPS receiver) and an energy supplement module (such as solar panels).
